# Intact mass analysis reveals the novel O-linked glycosylation on the stalk region of PD-**1** protein

**DOI:** 10.1038/s41598-023-36203-3

**Published:** 2023-06-14

**Authors:** Phanthakarn Tit-oon, Arisa Wonglangka, Klaichan Boonkanta, Mathuros Ruchirawat, Mayuree Fuangthong, Ram Sasisekharan, Amnart Khongmanee

**Affiliations:** 1 Translational Research Unit, Chulabhorn Research Institute, Bangkok, 10210 Thailand; 2 Center for Biologics Research and Development, Chulabhorn Research Institute, Bangkok, 10210 Thailand; 3 Center of Excellence On Environmental Health and Toxicology (EHT), OPS, MHESI, Bangkok, Thailand; 4 Program in Applied Biological Sciences, Chulabhorn Graduate Institute, Bangkok, 10210 Thailand; 5 Koch Institute for Integrative Cancer Research, Massachusetts Institute of Technology, Cambridge, MA 02139 USA; 6 Department of Biological Engineering, Massachusetts Institute of Technology, Cambridge, MA 02139 USA

**Keywords:** Glycoproteins, Glycosylation, Membrane proteins, Cancer immunotherapy, Lymphocyte activation

## Abstract

Programmed cell death protein 1 (PD-1) is a key receptor in the immune checkpoint pathway and has emerged to be a promising target for cancer therapy. PD-1 consists of an intracellular domain followed by a transmembrane domain that is connected to the extracellular domain by the stalk region. Although the PD-1 structure has been studied for more than two decades, the posttranslational modification of this protein has been incompletely characterized. In this study, we identified the previously undescribed modification sites of O-linked glycan on the stalk region of PD-1 protein using O-protease digestion coupling with intact mass analysis. The result indicates that T153, S157, S159, and T168 are modified by sialylated mucin-type O-glycan with core 1- and core 2-based structures. This study provides both information on potential novel modification sites on the PD-1 protein and an attractive method for identifying O-linked glycosylation using a specific enzyme and intact mass analysis.

## Introduction

Programmed cell death protein 1 (PD-1) is a transmembrane immunoreceptor protein that is expressed at the cell surface of T lymphocytes (T cells)^1^. PD-1 interacts with two ligands—programmed death 1 ligand-1 (PD-L1)^2,3^ and programmed death 1 ligand-2 (PD-L2)^4,5^—on the cell surface of normal and cancer tissues. The binding of PD-1 and its ligands downregulates the antigen receptor signaling of the effector T cells to prevent unnecessary immune responses, thereby playing a crucial role in autoimmunity, allergy, transplantation immunity, infectious immunity, and cancer immunity^1,6^. Thus, advancing understanding of the structure and function of PD-1 is undoubtedly valuable.

PD-1 consists of an IgV-like domain, a transmembrane domain, a cytosolic domain, and a stalk region that separates the IgV domain from the plasma membrane^7,8^. Protein modification in the UniProt database (ID: Q15116) displays four N-linked glycosylation in the IgV-like domain and two tyrosine phosphorylation in the cytosolic domain of human PD-1 but no post-translational modification data in the stalk region. However, when using the NetOGlyc4.0 model^9^ to predict O-glycosylation of the PD-1 protein, certain serine, and threonine residues in the stalk region of PD-1 are potential modification sites for O-glycans.

Mass spectrometry has emerged as a powerful tool for the glycosylation analysis of proteins^10^. However, most techniques have employed mass spectrometry for the analysis of purified glycans or glycopeptide. Yang et al. demonstrated that O-protease enzyme (OpeRATOR) is an effective enzyme for the study of O-glycosylation via mass spectrometry analysis of glycopeptide^11^. We propose a new method that combines this enzyme digestion with intact mass analysis to study O-glycosylation of the PD-1 protein. Herein, we provide evidence that there is O-linked glycosylation on the stalk region of PD-1. We measure the change in molecular weight (MW) of PD-1 proteins treated with various enzymes specific to N-linked and O-linked glycosylation, including PNGase F, sialidases, and OpeRATOR, using SDS-PAGE and intact mass analysis. We also mutate the candidate serine and threonine residues to identify the attachment sites of O-linked glycosylation.

## Results

### The evidence of O-linked glycans on the PD-1 stalk region from enzymatic digestion and SDS-PAGE analysis

**Figure 1:**
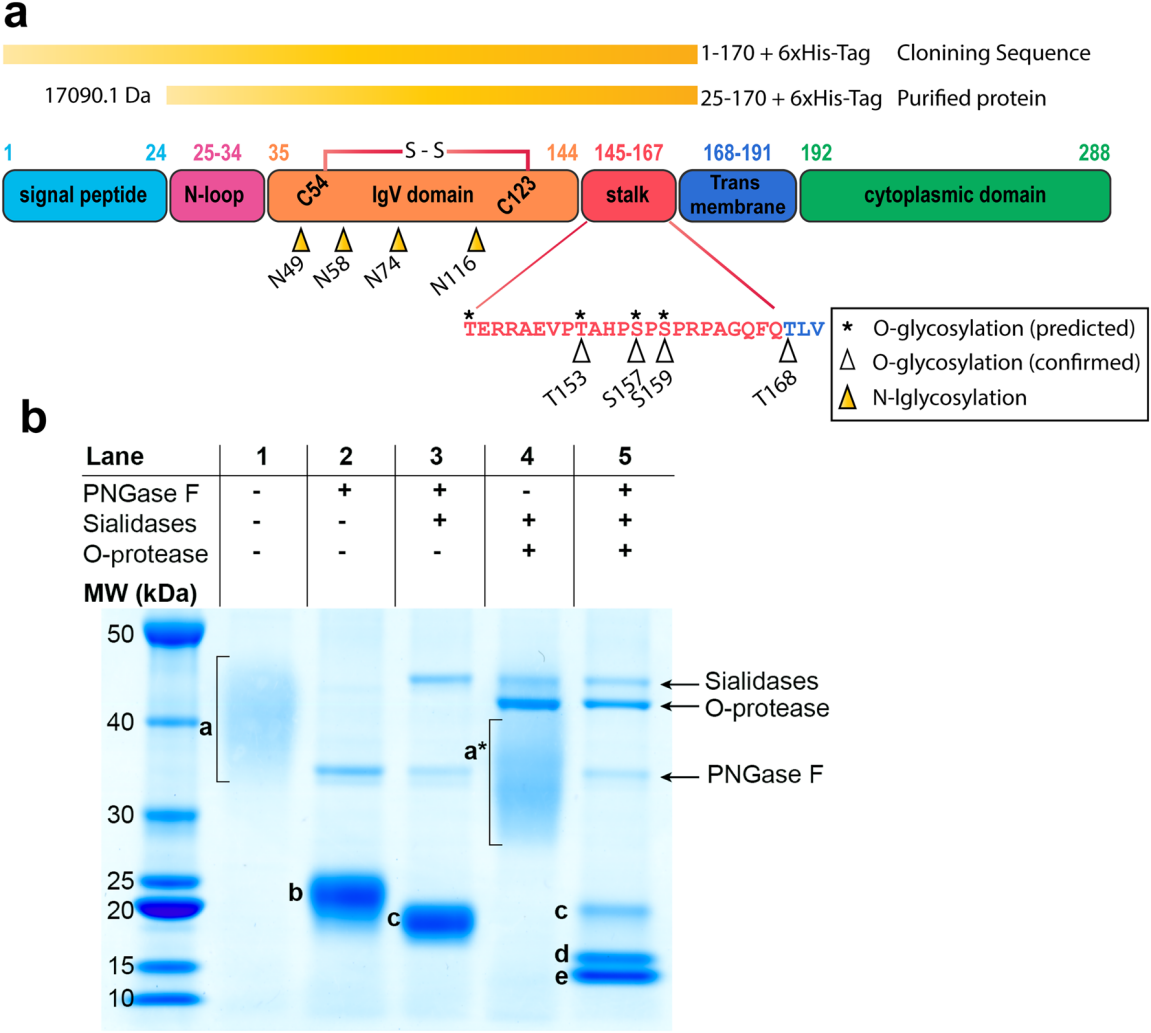
Overview structure of PD-1 protein, cloning sequence, and SDS-PAGE result. (**a**) Schematic of PD-1 protein showing different domains, position of amino acid in each domain, disulfide bond location, N-linked glycosylation, predicted and identified O-linked glycosylation, range of cloning sequence, and range of purified recombinant protein with MW calculated. Amino acid positions corresponded to UniProt (Q15116). (**b**) SDS-PAGE analysis of recombinant PD-1 protein. Untreated (Lane 1), treated with PNGase F (Lane 2), treated with PNGase F and sialidases (Lane 3), treated with sialidases and O-protease (Lane 4), and treated with the 3 enzymes (Lane 5). Band a–e are described in text; bands correspond to PNGase F, sialidases, and O-protease enzymes are indicated. Band a* corresponds to PD-1 protein treated with sialidases and O-protease. The gel was scanned using an EPSON Expression 11000XL scanner, and images were exported using SilverFast 8.0 software. The original uncropped image of gel in this figure is provided in supplementary Fig. S1.

We hypothesized that the stalk region of PD-1 contains O-linked glycosylation. To prove this hypothesis, the extracellular domain, including the stalk region of the PD-1 protein (amino acids 1–170; Fig. 1a), was expressed in HEK293 cells and purified for further analysis. The purified PD-1 protein was expected to contain amino acids 25–170 without the signal peptide (amino acids 1–24; Fig. 1a). The purified recombinant PD-1 protein was then subjected to digestion using a series of enzymes to remove glycan modifications. For the untreated recombinant PD-1 protein, it appeared as a broad band on SDS-PAGE with a molecular weight (MW) ranging from approximately 35 to 45 kDa (Fig. 1b, lane 1, band a), while the MW of the naked protein was only 17,090.1 Da. This is consistent with the presence of known N-linked glycans on the PD-1 protein^7^. When the PD-1 protein was treated with PNGase F enzyme to remove the N-linked glycans, the apparent MW of the PD-1 protein on SDS-PAGE significantly reduced to 20–25 kDa (Fig. 1b, lane 2, band b). This result indicated that N-linked glycans account for about 15–20 kDa of the total MW of the PD-1 protein. Although the removal of N-linked glycans narrowed the band of the protein that appeared on SDS-PAGE, this band was still a broad and larger than expected naked protein (17 kDa). The result suggested that other modifications exist on the protein. An additional digestion of the PNGase F-treated recombinant PD-1 with sialidases to remove terminal sialic acid in the glycan structure further reduced the size of the protein to about 18–19 kDa; however, it was still larger than the naked protein (Fig. 1b, lane 3, band c). These results strongly suggested the presence of sialylated O-linked glycans on the PD-1 protein. To verify the presence of O-linked glycans, O-protease enzyme (OpeRATOR), which cuts the peptide bond at N-terminus of serine or threonine with core 1 O-glycan modification, was employed. The recombinant PD-1 treated with PNGase F, sialidases, and O-protease showed two major bands on SDS-PAGE (Fig. 1b, lane 5, bands d and e), indicating the presence of O-linked glycan. The MW of band c was similar to the PD-1 protein that was treated with PNGase F and sialidases (Fig. 1b, lane 3, band c), suggesting incomplete digestion. Treatment of the recombinant PD-1 protein with only sialidases and O-protease yielded a broad band (Fig. 1b, lane 4, band a*) with slightly smaller MW compared with band a. This result indicated that O-protease could cut the protein even when N-linked glycans are present.

### Confirmation of O-linked glycan on the PD-1 protein using intact mass analysis

**Figure 2:**
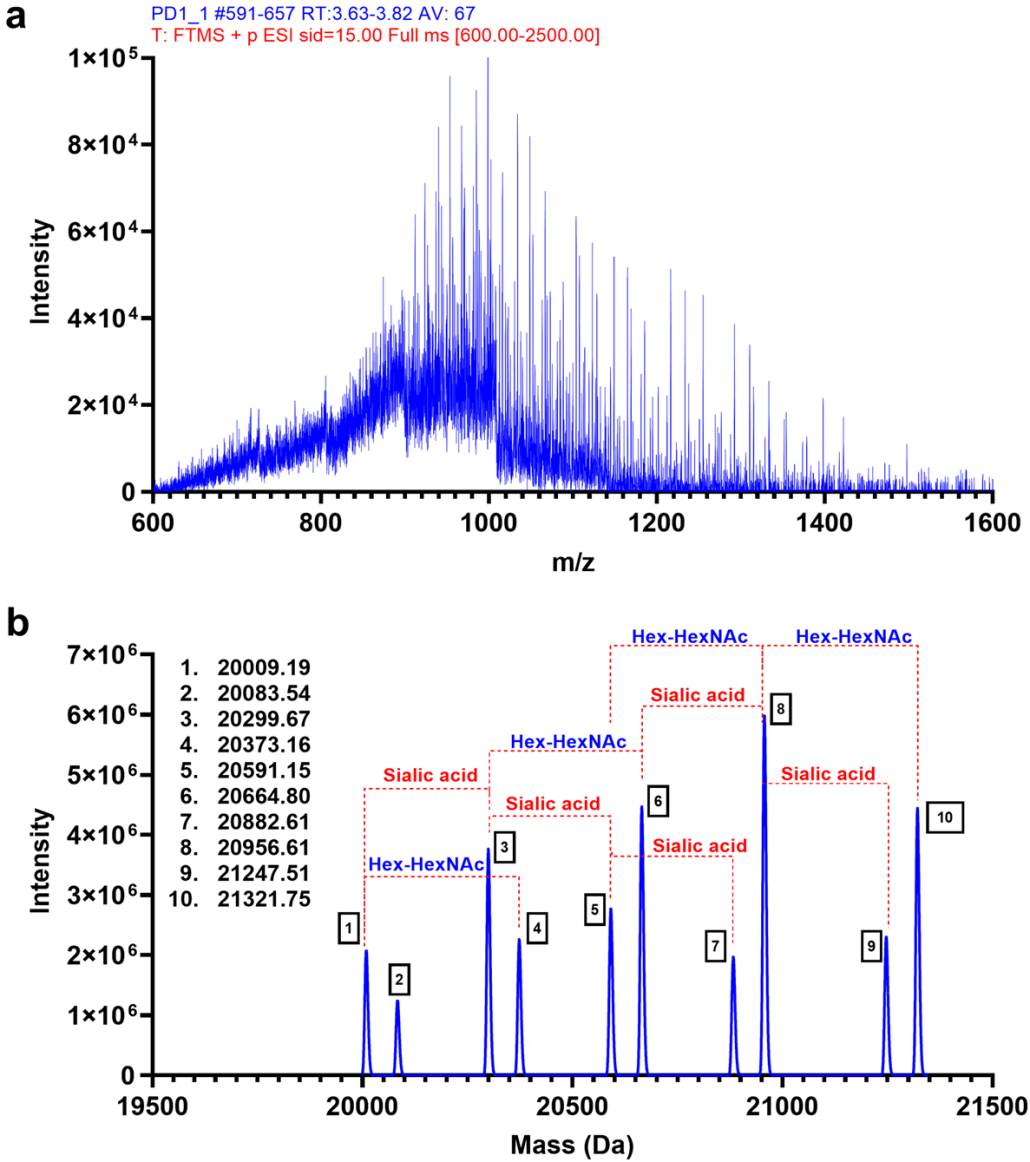
Intact mass analysis of N-linked removed recombinant PD-1. (**a**) An average raw mass spectrum of the intact protein over eluted retention time that was used for protein deconvolution. (**b**) A representative deconvolution mass of the N-linked removed recombinant PD-1; delta mass to approximately 365 Da was labeled as Hex-HexNAc, and to approximately 291 Da was labeled as sialic acid. Graphs were generated in GraphPad Prism version 9.4.1 for Windows (https://www.graphpad.com/) using original data obtained from Biopharma Finder 3.0 and Qual Browser, Thermo Xcalibur 3.1.66.10, as shown in supplementary Figs. S2, 3.

A more accurate MW determination of the recombinant PD-1 protein treated with various enzymes was performed using intact mass analysis. The raw mass spectrum from the recombinant PD-1 protein without N-linked glycans was complex due to the presence of various glycoforms and terminal sialic acid in the structure that could hinder overall ionization (Fig. 2a). However, deconvoluted masses ranging from 20 to 22 kDa in this sample were consistent with the prior SDS-PAGE result (Fig. 1b, lane 2, band b). Interestingly, the delta mass of 291 Da (sialic acid structure) and 365 Da (Hex-HexNAc structure) were observed between those species of identified intact masses, which indicated that other glycan structures still existed on the protein (Fig. 2b).

**Figure 3:**
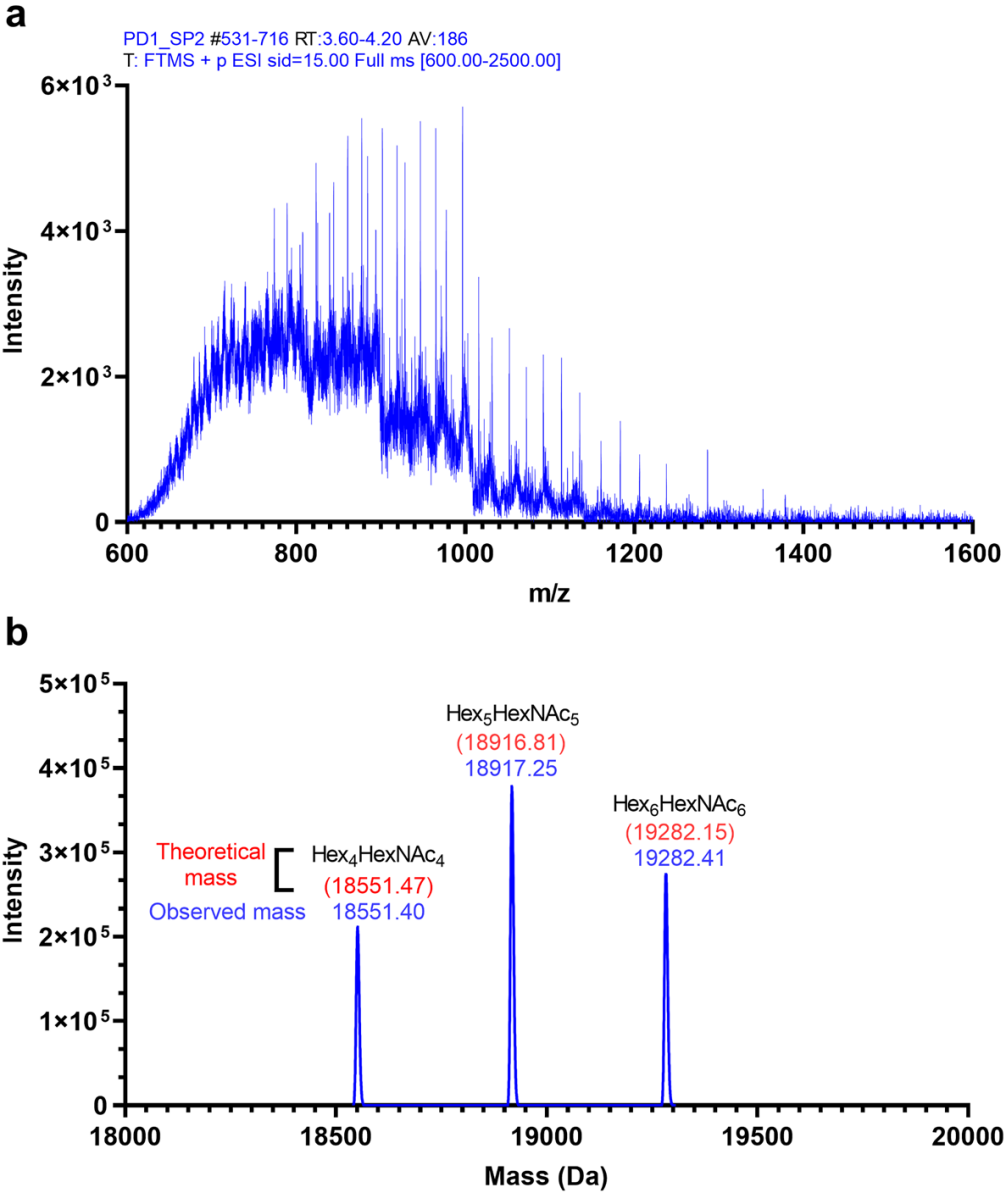
Intact mass analysis of N-linked removed recombinant PD-1 treated with sialidases. (**a**) An average raw mass spectrum of the intact protein over eluted retention time that was used for deconvolution. (**b**) A representative deconvolution mass of the sample; theoretical mass with the proposed glycan component was calculated for each deconvoluted glycoform (red), compared with observed mass (blue). Graphs were generated using GraphPad Prism version 9.4.1 for Windows (https://www.graphpad.com/) using original data obtained from Biopharma Finder 3.0 and Qual Browser, Thermo Xcalibur 3.1.66.10, as shown in supplementary Figs. S4, 5.

N-linked glycan-removed PD-1 protein was treated with sialidases to reduce the complexity and increase the ionization of the sample. The intact mass analysis result of this sample was simpler than the previous sample (Fig. 3a). Three distinct species of protein with different MW were identified (Fig. 3b). The differences between the observed mass and the calculated mass of naked protein (17,090.1 Da) were used to estimate the possible composition of the glycans attached to the protein (Fig. 3b). The glycan structures, which consisted of Hex_4_HexNAc_4_, Hex_5_HexNAc_5_, and Hex_6_HexNAc_6_, were still attached to the PD-1 protein, resulting in MWs of 18,551.40 Da, 18,917.25 Da, and 19,282.41 Da, respectively. Using this result, we could conclude that multiple O-linked glycans exist on the PD-1 protein. However, the site of the modifications was not known.

### Identification of T153 and S157 as O-linked glycosylation sites on PD-1 protein

**Figure 4:**
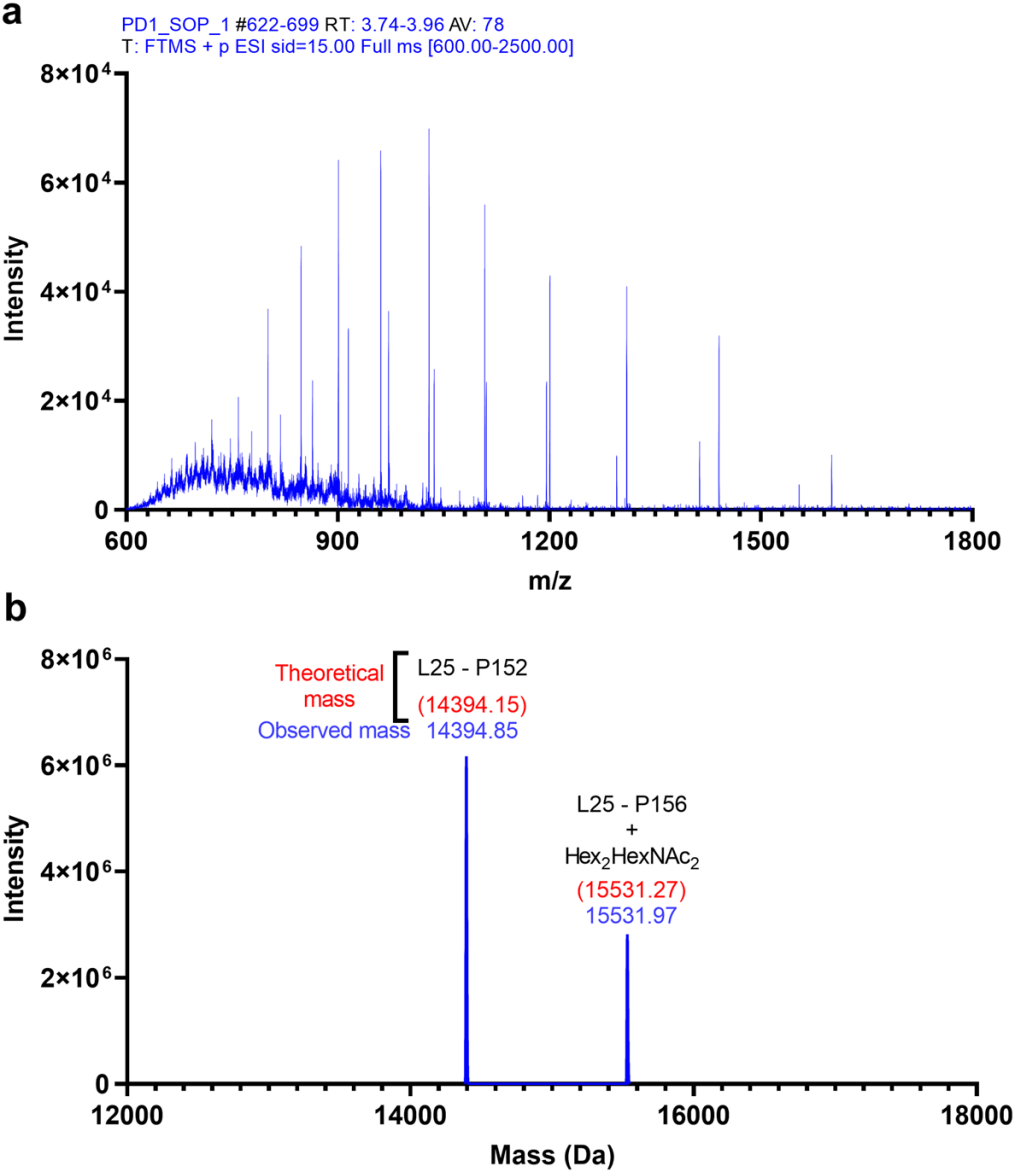
Intact mass analysis of N-linked removed recombinant PD-1 treated with sialidases then digested with O-protease enzyme. (**a**) An average raw mass spectrum of the intact protein over eluted retention time that was used for deconvolution. (**b**) A representative deconvolution mass of the sample; theoretical mass was calculated for each deconvoluted truncated protein (red), compared with observed mass (blue). Graphs were generated using GraphPad Prism version 9.4.1 for Windows (https://www.graphpad.com/) using original data obtained from Biopharma Finder 3.0 and Qual Browser, Thermo Xcalibur 3.1.66.10, as shown in supplementary Figs. S6, 7.

To predict the sites of the O-linked glycans on the PD-1 protein, NetOGlyc 4.0 Server was used. A total of four possible O-linked glycan modification sites were predicted with scores greater than 0.5 (Table 1). Four of the predicted sites were in the stalk region (i.e., T145, T153, S157, and S159), whereas the other site (S273) was in the cytosolic domain. To confirm this prediction, we performed intact mass analysis of the PD-1 protein after it had been treated with PNGase F, sialidases, and O-protease. O-protease is an enzyme that specifically cut protein at the N-terminus of serine or threonine residues modified with core 1 O-GalNAc glycan. The mass analysis result of this sample was less complex than that of the previous samples (Fig. 4a). Only two distinct species of protein were identified with MWs of 14,394.85 Da and 15,531.97 Da (Fig. 4b), which is consistent with the two bands observed on SDS-PAGE (Fig. 1b, lane 5, bands d and e). This result strongly indicates the presence of O-linked glycan on the PD-1 protein.

**Table 1:** Prediction of the possible O-linked glycosylated sites on PD-1 protein using NetOGlyc 4.0 Server.

Amino acid position	Domain	Predicted score
T145	Stalk	0.767
T153	Stalk	0.967
S157	Stalk	0.865
S159	Stalk	0.898
S273	Cytoplasmic domain	0.671

All positions with a score greater than 0.5 are listed in the table.

In conjunction with the predicted O-linked glycosylated sites from NetOGlyc4.0 server, which suggested that threonine position 153 (T153) is modified with O-linked glycan, we found that O-protease was cut protein at this position. If the O-protease cut the recombinant PD-1 protein at the N-terminus of T153, the average MW of the truncated protein (amino acids 25–152) would be equal to 14,394.15 Da. This closely matched the main observed mass (14,394.85 Da), with the delta mass less than 1 Da (Fig. 4b). Therefore, T153 is one of the O-linked glycosylated sites on the stalk region of the PD-1 protein. The other observed molecular mass of 15,531.97 Da is consistent with the O-linked glycan at S157. If T153 were modified by the core 2 (Hex_2_HexNAc_2_) O-linked glycans that O-protease was unable to digest, and the protein were cut at the N-terminus of serine position 157 (S157), the average mass of this truncated O-linked glycosylated PD-1 protein (amino acid 25–156 with Hex_2_HexNAc_2_) would be equal to 15,531.27 Da. This calculated MW perfectly matched the observed mass of 15,531.97 Da, with the delta mass less than 1 Da (Fig. 4b). Therefore, this result indicated that S157 was another O-linked glycosylated site on the stalk region of the PD-1 protein. Thus, these results strongly support the prediction that T153 and S157 are O-glycan modification sites on the stalk region of PD-1. In addition, the in-gel tryptic digestion of bands a, b, d, and e in Fig. 1b also confirms that the O-protease enzyme was cleaved at the N-terminus of T153 and S157 (see supplementary Figs. S17–21).

### Confirmation of T153, S157, S159, and T168 as O-linked glycosylation sites on the PD-1 protein by alanine mutation

**Figure 5:**
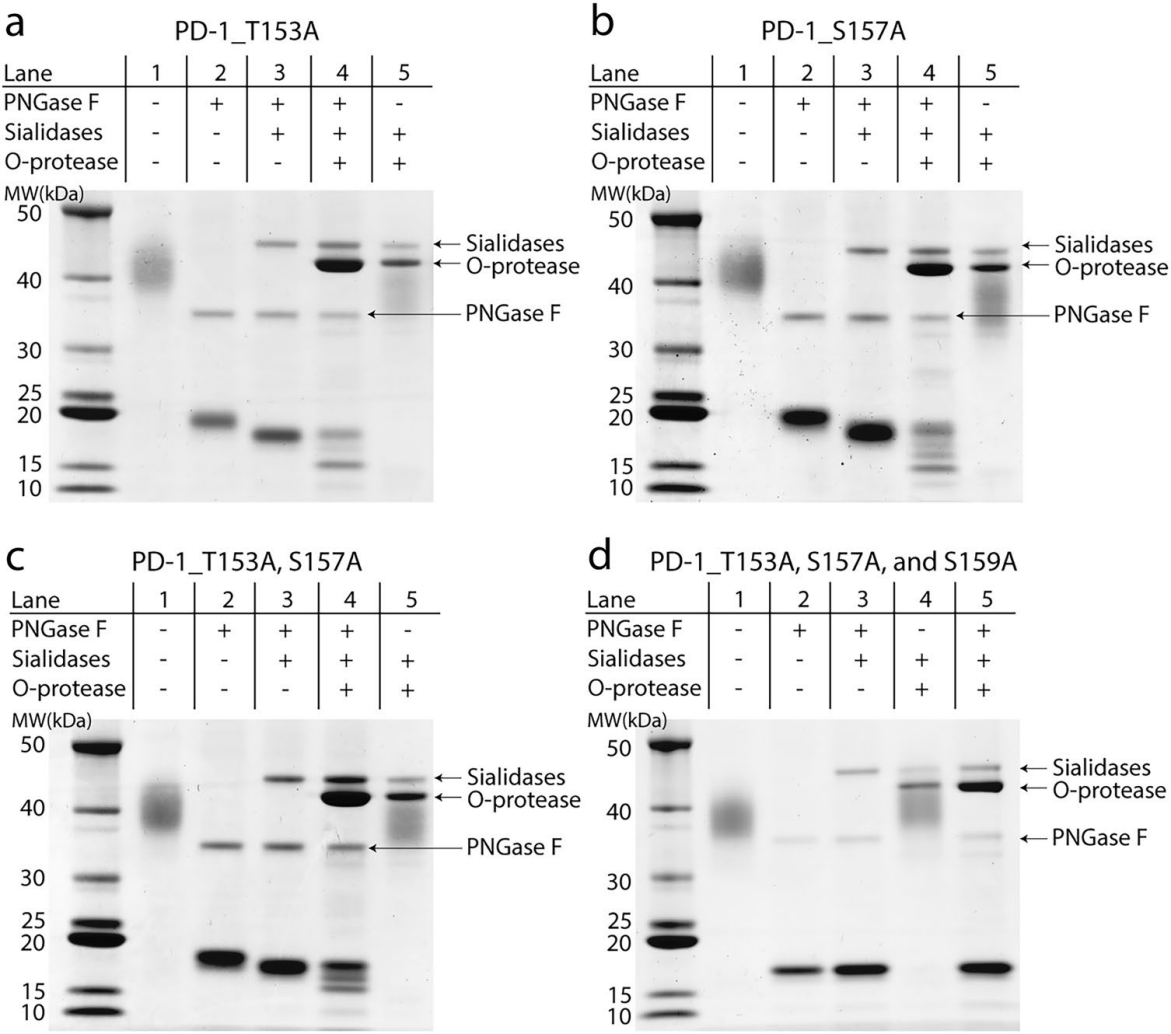
SDS-PAGE analysis of four alanine mutation PD-1 proteins that were treated with PNGase F, sialidases, and O-protease in various combinations. (**a**) PD-1 with T153A mutation, (**b**) PD-1 with S157A mutation, (**c**) double mutation of PD-1 at positions T153A and S157A, and (**d**) triple mutation of PD-1 at positions T153A, S157A, and S159A. The gels were scanned using EPSON Expression 11000XL scanner, and the images were exported using SilverFast 8.0 software. The original uncropped image of the gel in this figure is provided in supplementary Figs. S8–S10.

**Figure 6:**
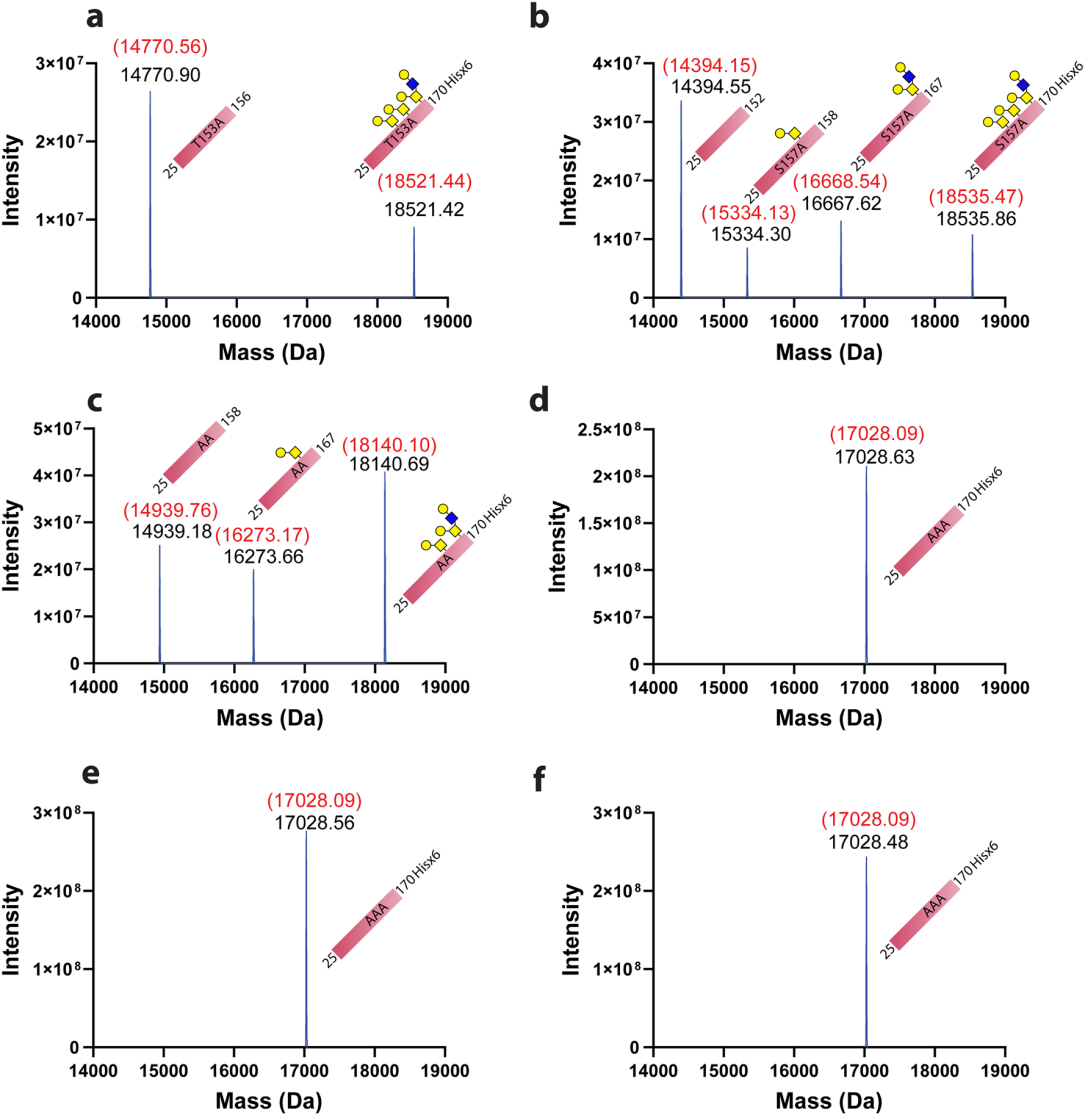
Intact mass analysis of four alanine mutation PD-1 proteins that were treated with PNGase F, sialidases, and O-protease in combination. (**a**) PD-1 with T153A mutation, (**b**) PD-1 with S157A mutation, (**c**) double mutation of PD-1 at positions T153A and S157A, and (**d**) triple mutation of PD-1 at positions T153A, S157A, and S159A treated with PNGase F, (**e**) triple mutation of PD-1 at positions T153A, S157A, and S159A treated with PNGase F and sialidases, and (**f**) triple mutation of PD-1 at positions T153A, S157A, and S159A treated with PNGase F, sialidases, and O-protease. MW shown in red is based on calculations with modifications, as described in detail in the corresponding text. MW shown in black represents deconvoluted mass from experimental data. Graphs were generated using GraphPad Prism version 9.4.1 for Windows (https://www.graphpad.com/) using original data obtained from Biopharma Finder 3.0, as shown in supplementary Figs. S11–16.

To confirm that T153 and S157 are the sites for O-linked glycosylation on PD-1 protein, we constructed PD-1 proteins carrying the T153A or S157A mutation. We performed the same enzyme digestion series on the mutated PD-1 proteins as had previously been done on the wild-type proteins. PNGase F and sialidases were used to reduce the complexity of the samples due to the N-linked glycosylation and sialic acid capping on the O-linked glycan structure. Digested proteins were analyzed at each step by SDS-PAGE and intact mass analysis. O-protease digestion of PNGase F and sialidases treated with PD-1 protein carrying the T153A mutation resulted in a prominent single band on SDS-PAGE at 15 kDa, instead of 2 bands on the wild-type protein (Fig. 5a, lane 4). Note that the band at around 18–19 kDa was predicted to be incomplete digestion of O-protease, as the band comigrated with the protein observed in the reaction with PNGase F and sialidases alone. Intact mass analysis of the T153A mutation of the PD-1 protein that was treated with PNGase F, sialidases, and O-protease revealed a prominent MW of 14,770.90 Da (Fig. 6a). This observed mass closely matched a fragment of PD-1 with T153A mutation that was cut by O-protease at S157, which was calculated to be 14,770.56 Da. This result confirmed that Hex_2_HexNAc_2_ glycan that modified the previously observed fragment of 15,531.97 Da in Fig. 4b was attached to T153. Therefore, mutating this T153 to alanine removed the Hex_2_HexNAc_2_ modification and yielded an MW of 14,770.90 Da. The intact mass analysis of this sample also revealed an undigested fragment of this mutant with an MW of 18,521.42 Da, which perfectly matched the calculated MW of PD-1 with the T153A mutation with Hex_4_HexNAc_4_ (18,521.44 Da).

For PD-1 protein with S157A mutation, PNGase F, sialidase, and O-protease digestion resulted in several bands of protein on SDS-PAGE, but the most notable band appeared at slightly lower than 15 kDa (Fig. 5b, lane 4). Intact mass analysis of this sample revealed four fragments of PD-1 protein with an MW of 14,394.55, 15,334.30, 16,667.62, and 18,535.86 Da (Fig. 6b). The higher abundance fragment had an MW of 14,394.55 Da, indicating the digestion of O-protease at O-glycosylation site T153, as previously observed in Fig. 1b, lane 5, band e. The lower abundance fragment with an MW of 15,334.30 Da matched the calculated mass of PD-1 with S157A mutation plus Hex_1_HexNAc_1_ glycan structure that was cut by O-protease at S159 (15,334.13 Da). This result provided evidence that S159 was modified by O-glycans. Another fragment with an MW of 16,667.62 Da that was found in this sample closely matched the calculated mass of PD-1 protein carrying S157A mutation plus Hex_2_HexNAc_2_ glycan structure that was cut by O-protease at T168 (16,668.54 Da). This result indicated that T168 in the stalk region of PD-1 contained O-linked glycosylation. Interestingly, the predicted score for the O-linked glycosylated site of T168 using NetOGlyC 4.0 was lower than the cut-off value^9^. The fourth fragment that was found in this sample had an MW of 18,535.86 Da, which closely matched the undigested mass of PD-1 with S157A mutation that was modified by the Hex_4_HexNAc_4_ glycan structure (18,535.47 Da).

The double mutation of PD-1 at T153A and S157A treated with a combination of PNGase F, sialidases, and O-protease revealed two notable bands on SDS-PAGE (Fig. 5c, lane 4). Intact mass analysis of this O-protease-treated sample revealed three protein fragments with an MW of 14,939.18, 16,273.66, and 18,140.89 Da (Fig. 6c). The small fragment with an MW of 14,939.18 Da matched the calculated mass of this double mutation of PD-1 that was cut by O-protease at S159 (14,938.76 Da). This result was consistent with the data from the S157A mutation of PD-1 that showed S159 had been modified by O-glycans. The second fragment of this sample, with an MW of 16,273.66 Da, matched the calculated mass of this protein plus Hex_1_HexNAc_1_ glycan structure that was cut at T168 (16,273.17 Da). This result indicated that T168 on the stalk region of PD-1 was modified by O-glycans. The third fragment with the MW of 18,140.69 Da matched the calculated mass of undigested double mutation PD-1 at position T153A and S157A carrying Hex_3_HexNAc_3_ glycan structure (18,140.10 Da).

The triple mutation of PD-1 at T153A, S157A, and S159A that was treated with (i) PNGase F alone, (ii) PNGase F and sialidases, and (iii) PNGase F, sialidases, and O-protease revealed only a single band on SDS-PAGE, as shown in lanes 2, 3, and 5, respectively (Fig. 5d). Intact mass analysis of these three samples showed fragments of protein with an MW of 17,028.63, 17,028.56, and 17,028.48 Da (Fig. 6d–f, respectively). These MWs were closely matched to the calculated mass of triple-mutation PD-1 without glycosylation that equaled 17,028.09 Da. Interestingly, this result did not indicate that T168 had been modified by O-glycans, as observed from the S157A mutation and T153A with S157A double-mutation. Altogether, the results indicated that T153, S157, S159, and possibly T168 on the stalk region of PD-1 were modified by O-glycans.

## Discussion

In this study, we identified novel O-glycosylation sites within the stalk region of PD-1 protein at positions T153, S157, S159, and possibly T168. To the best of our knowledge, there has been no report related to O-linked modifications on PD-1 protein, although PD-1 has been in the spotlight of cancer immunotherapy as a promising drug target for more than two decades^1^. Information from the UniProt database (Q15116) indicated that the PD-1 protein was modified by N-linked glycans but not O-linked. It is possible that the identified O-linked glycans in this study were hidden in the region close to the transmembrane domain of the protein. Moreover, the extracellular region of PD-1 was also modified by four N-linked glycosylations, which increase the heterogeneity and complexity of the protein. Altogether, this might be the reason why these novel O-linked glycosylations have never been found.

Even though the results from this study confirm that the recombinant PD-1 protein was modified by O-linked glycans, the function of surface glycoprotein modifications at the cellular level has yet to be proved. Chapman et al. demonstrated that O-glycan modification on the stalk region of the 75-kDa neurotrophin receptor (p75^NTR^) plays a role in the binding constant of the receptor^12^. Yeaman C et al. also reported that O-glycosylation on the stalk region is required for apical sorting of p75^NTR^ in polarized MDCK cells^13^. PD-1 is mainly expressed on T cells that become polarized during migration^14^. Thus, it is possible that O-linked glycosylation on the stalk region of PD-1 could facilitate the correct sorting of the receptor during the migration of T cells. Klíma M et al. reported that the stalk region of death receptor 6 (DR6/TNFRSF21) is likely modified by mucin-type O-glycans, and deletion of the stalk region on DR6 also disrupts the expression of receptors on the cell surface^15^. Interestingly, the glycan structure that modified p75^NTR^ and DR6 was identified to be mucin-type O-GalNAc, which was found in the stalk region of PD-1 in this study. Other functions of the O-glycosylated stalk region could be protected from proteolysis^16^ or propagated signal transduction from ligand binding to the transmembrane and intracellular domain^12,17^. Among the possible functions of O-linked glycosylation on the stalk region of the PD-1 protein, we believe that the most likely function of these modifications is the steric interaction between O-glycans and the peptide backbone in the stalk region could form a rod-like structure and stretch the receptor on the cell surface^18,19^.

**Figure 7:**
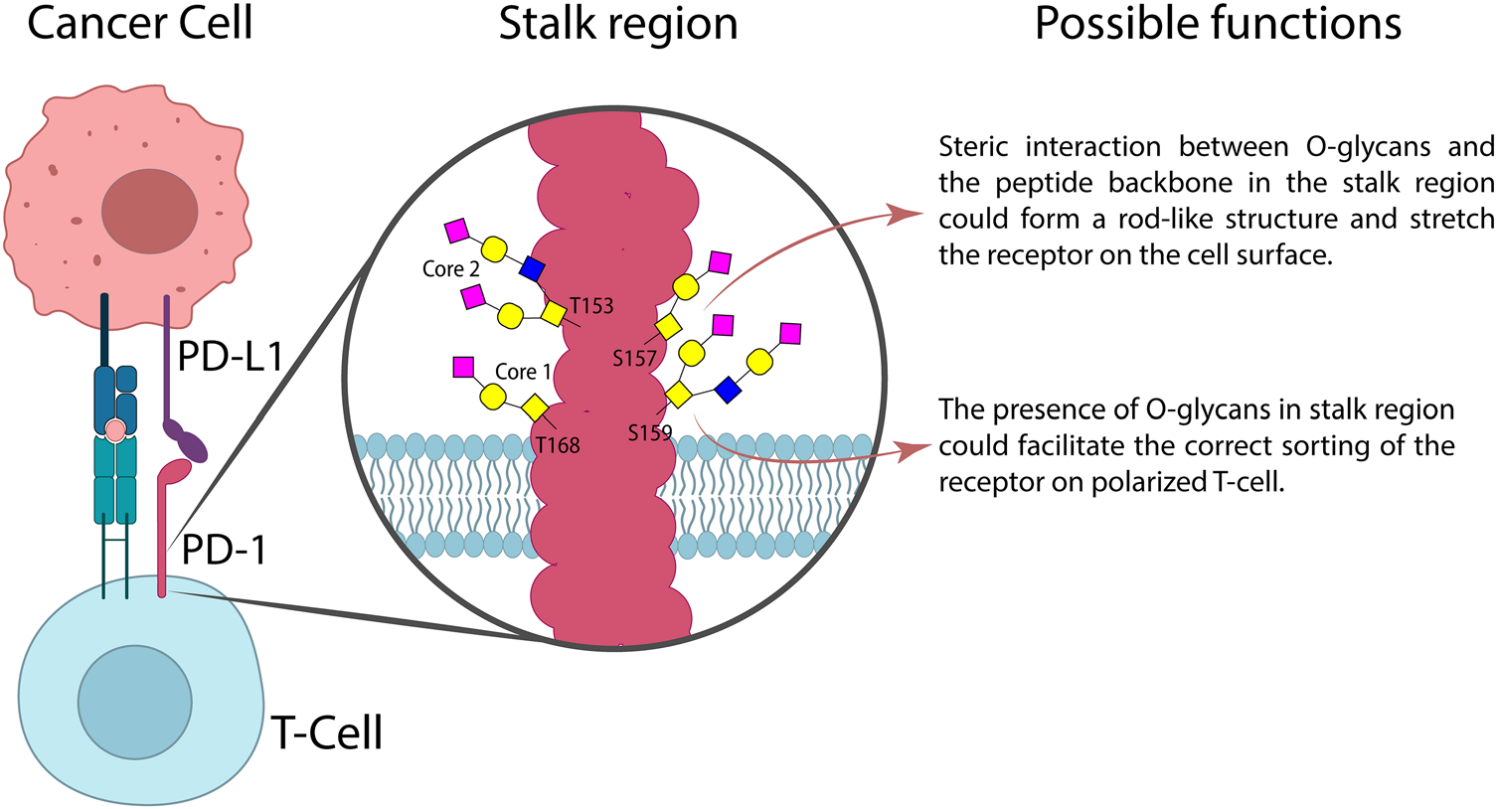
Summary of key finding in this study including structure of O-linked glycosylation in the stalk region of PD-1 and the proposed functions.

Liquid chromatography coupled with mass spectrometry (LC–MS) is an indispensable technique in protein glycosylation studies^20^. Typically, glycosylation studies using LC–MS can analyze on three levels: (i) for released glycans for structural analysis, (ii) for glycans removed from peptides for the identification of glycosylation sites, and (iii) for intact glycopeptides, which provide both glycan structures and modification sites^11^. Intact mass analysis is rarely used to study protein glycosylation, except for the case of biologic drugs^21^. In this study, we demonstrated for the first time that intact mass analysis in combination with O-protease digestion can identify O-linked glycosylated sites on the PD-1 protein at T153 and S157. We further confirmed this discovery with alanine mutation, which was guided by the bioinformatics tool, leading to the discovery that S159 and T168 are also modified by O-linked glycans. The specificity of this O-protease is the key for this approach, as it allows us to pinpoint specific O-linked glycosylated sites on the stalk region of the PD-1 protein. Additionally, the fact that O-protease prefers the digestion of core 1 rather than core 2 structure provided an opportunity to identify more than one modification site on the same protein. Summary of possible O-linked glycan structures, modification sites, and proposed functions were shown in Fig. 7. It is notable that three-quarters of the predicted O-linked glycosylation on the PD-1 protein by NetOGlyc 4.0 server were experimentally confirmed. It is undoubted that this bioinformatics tool provides valuable information for novel O-linked glycosylation identification^9^.

In general, electron transfer dissociation is the method of choice for detecting O-glycosylation sites in glycopeptides^22^. However, not everyone has the privilege to access such a prestigious and expensive tool. The method we have presented in this study adds a new alternative for identifying O-glycosylation sites and their structure using conventional high-resolution mass spectrometry. Nevertheless, this technique could significantly simplify O-linked glycosylated protein characterization, but its benefit is only limited to the mucin-type O-linked glycosylated protein that the O-protease derived from *Akkermansia muciniphila* is able to digest^23^. Even though there is this limitation to this approach, it still provides clues that the mucin-type glycan structures form the modification on the stalk region of PD-1 protein, as was discovered in this study.

In summary, this study demonstrated a novel method to characterize O-linked glycosylated protein using O-protease digestion and intact mass analysis. The results suggest that the stalk region of PD-1 at T153, S157, S159, and T168 are modified by sialylated O-glycan with core 1–and core 2–based structures. This is a generalized approach that can readily be applied to other important proteins.

## Methods

### Reagents and chemicals

O-protease (OpeRATOR) and sialidase (SialEXO) enzymes were purchased from Genovis. Rapid PNGase F enzyme was purchased from New England BioLabs. Trypsin enzyme (Trypsin Gold, Mass Spectrometry Grade) and ProteaseMAX™ Surfactant (Trypsin Enhancer) was purchased from Promega. LC/MS-grade acetonitrile was purchased from Pierce. LC/MS-grade formic acid (FA) was obtained from Fisher Chemical. All aqueous solutions were prepared using LC/MS-grade water (Thermo Fisher Scientific). Sodium phosphate, sodium chloride, and imidazole were purchased from Sigma. Ammonium bicarbonate, 1,4-Dithiothreitol (DTT), and Iodoacetamide were purchase from Sigma-Aldrich.

### Construction of PD-1 expression vector

PD-1 wild-type and mutant genes—T153A, S157A, double mutation (T153A and S157A), and triple mutation (T153A, S157A, and S159A)—with Kozak consensus sequence and hexa-histidine tags were synthesized by ATUM and Genscript. The PD-1 gene was then cloned into pcDNA3.1(+) (Invitrogen) at *Bam*HI and *Eco*RV sites. The recombinant plasmids were confirmed using restriction enzyme digestion and DNA sequencing. The plasmids were transformed into MAX Efficiency DH5αF'IQ Competent Cells (Invitrogen) and purified using PureLink HiPure Plasmid Maxiprep kit (Invitrogen). The plasmids were stored at − 20 °C.

### Cell culture and PD-1 protein expression

Expression of His-tagged PD-1 wild-type protein and its alanine mutants was carried out using FreeStyle 293-F suspension cells (Invitrogen). Cells were grown in FreeStyle 293 Expression Medium (Invitrogen) and maintained at 37 °C, 80% humidity, and 8% CO_2_ in an orbital shaker at 130 rpm. Eighty milliliters of cell culture was transiently transfected with 100 µg of PD-1 plasmid using Polyethylenimine Max Transfection Reagent (PolySciences) in 150 mM sodium chloride solution. The culture medium was then harvested for 7 days after transfection.

### PD-1 protein purification

The supernatant was collected by centrifugation at 4000 RCF at 4 °C for 10 min and filtered through Acrodisc Syringe Filters with 0.2 µm Supor Membrane (Pall). Protein purification was performed using an ÄKTA pure FPLC system and 0.7 cm × 2.5 cm HisTrap High Performance column (GE Healthcare). The column was equilibrated using buffer A (20 mM sodium phosphate, 0.5 M sodium chloride, 30 mM imidazole, pH 7.4). Samples were injected into the equilibrated column at a flow rate of 1 mL/min, and the bound protein was eluted using linear gradient from 0 to 100% of buffer B (20 mM sodium phosphate, 0.5 M sodium chloride, 500 mM imidazole, pH 7.4) for 20 min. Elution was monitored using UV absorbance at 280 nm. Fractions were collected, and the buffer was exchanged to phosphate-buffered saline (PBS) using Amicon Ultra Centrifugation Filters (Merck).

### SDS-PAGE and staining

Protein samples (800 ng) were mixed with a sample buffer containing 0.1% β-mercaptoethanol and resolved in NuPAGE 4–12% Bis–Tris Protein Gels (Invitrogen) and electrophoresed in a XCell SureLock Mini-Cell Electrophoresis System (Thermo Fisher Scientific) using a constant voltage (120 V) at room temperature for 2 h. Protein bands were visualized using InstantBlue Protein Stain (Expedeon). The gels were then scanned with an EPSON Expression 11000XL scanner (Epson), and images were collected/exported using SilverFast 8.0 software (Epson).

### Enzymatic digestion

To remove N-linked glycans, 45 µg of recombinant PD-1 protein in PBS buffer was digested with Rapid PNGase F. The reaction was incubated at 50 °C for 10 min. To remove terminal sialic acid, 40 µg of recombinant PD-1 in PBS buffer was incubated with 40 units of SialEXO at 37 °C for 2 h. To cut the recombinant protein at O-linked glycosylation sites, 40 µg of recombinant PD-1 in PBS buffer was incubated with 40 units of SialEXO followed by 40 units of OpeRATOR at 37 °C for 18 h.

### Intact mass analysis

Intact mass spectral data were acquired using an Orbitrap Elite Hybrid Ion Trap-Orbitrap Mass Spectrometer (Thermo Scientific) coupled to an ACQUITY UPLC System (Waters). Buffer A was 0.1% formic acid in water and buffer B was 0.1% formic acid in acetonitrile. Approximately 3 µg of protein samples were loaded in an MAbPac RP Column (4 µm, 2.1 mm × 50 mm, Thermo Scientific) preconditioned with 20% buffer B. The first 2 min of gradient were maintained at 20% buffer B. The separation was attained using solvent gradient ramping from 20 to 65% buffer B over 4.5 min. Washing was done using 90% buffer B for 3.5 min. The column was then re-equilibrated with 20% buffer B for 2 min. The flow rate was set at 0.3 mL/min throughout all gradient steps. The column temperature was maintained at 80 °C throughout all gradient steps. The HESI-II ion source parameters were set as follows: spray voltage 4.00 kV, capillary temperature 300 °C, S-lens RF 70%, sheath gas flow 50 units, and auxiliary gas flow 15 units. The data acquisition parameters were set as follows: scan range m/z 600–2500, resolution 15,000, positive mode, FT Full scan AGC target 3 × 10^6^, fixed AGC mode, and enabled full-scan injection waveforms.

### Deconvolution of intact mass and predicted MW calculation

Intact mass spectra were deconvoluted using BioPharma Finder version 3.0 software (Thermo Scientific). The parameters for intact protein analysis were set as follows: the chromatogram m/z range was set to 600–2500, sensitivity was set to "High", chromatogram trace type was set to "TIC", source spectra method was set to "Average Over Selected Retention Time", deconvolution algorithm was set to "ReSpect", output mass range varied between 10,000 and 22,000 Da depending on each fragment, deconvoluted spectra display mode was set to "Isotopic Profile", deconvolution mass tolerance was set to 20.0 ppm, choice of peak model was set to "Intact Protein", target mass varied depending on each fragment, and other parameters were set to default. The average molecular weight of protein sequences with and without glycan structures was calculated using Agilent MassHunter Sequence Manager B.09.00 software (Agilent Technologies).

### In-gel digestion

Protein bands in SDS-PAGE were digested using trypsin following the ProteaseMAX surfactant protocol. Briefly, the specific protein band was excised and the gel was cut into small pieces, then washed with water. Destaining was carried out with 200 μL of methanol:50 mM NH_4_HCO_3_ (1:1 v/v) for 1 min, followed by dehydration in 200 μL of acetonitrile:50 mM NH_4_HCO_3_ (1:1 v/v) for 5 min. Subsequently, 200 μL of 100% acetonitrile was added and after 30 s of incubation, the acetonitrile was discarded and the gels were dried in a vacuum centrifuge. Reduction of the dried gels was performed using 25 mM DTT in 50 mM NH_4_HCO_3_ at 56 °C for 20 min, followed by alkylation of free thiols with 55 mM iodoacetamide in 50 mM NH_4_HCO_3_ at room temperature in the dark for 20 min. The gels were washed twice with 400 μL of water and dehydrated again with 50% and 100% acetonitrile in 50 mM NH_4_HCO_3_. After discarding the supernatant, the gels were dried in a vacuum centrifuge. Rehydration and tryptic digestion of the dried gels was performed using 20 μL of 12 ng/μL trypsin in 0.01% ProteaseMAX Surfactant:50 mM NH_4_HCO_3_ for 10 min, followed by addition of 30 μL of 0.01% ProteaseMAX Surfactant:50 mM NH_4_HCO_3_. After gentle mixing, the reactions were incubated at 37 °C for 2 h. The reaction tubes were then centrifuged at 12,000×*g* for 10 s to collect the condensate and the gel pieces were discarded. The supernatant was transferred into a new tube and mixed with formic acid to a final concentration of 0.5% in order to inactivate trypsin. Prior to LC–MS/MS analysis, the samples were filtered through a 0.2 µm membrane Nanosep centrifugal devices (PALL).

### LC–MS/MS analysis

The LC–MS/MS data were obtained using an Orbitrap Elite Hybrid Ion Trap-Orbitrap Mass Spectrometer (Thermo Scientific). The mass spectrometer was operated using XCalibur software (Thermo Scientific). Peptides were separated using the UltiMate3000 RSLCnano System (Thermo Scientific) with a gradient of buffer A (0.1% formic acid in water) and buffer B (0.1% formic acid in 95% acetonitrile in water). One microliter of peptide sample was loaded onto an EASY-Spray Column ES802A Rev.2 (Thermo Scientific) that was preconditioned with 2% buffer B. The initial 3 min of the gradient were maintained at 3% buffer B, followed by a solvent gradient ramping from 2 to 40% buffer B over 60 min, and then ramping to 95% buffer B in 10 min. Washing was performed with 95% buffer B for 15 min, and the column was re-equilibrated with 2% buffer B for 32 min, resulting in a total runtime of 120 min per injection. The flow rate was set at 0.3 µL/minute for all gradient steps, and the column temperature was maintained at 35 °C. The NSI source parameters were set as follows: spray voltage 1.9 kV, capillary temperature 275 °C, S-lens RF 60%, and sweep gas flow rate of 5 units. Data acquisition parameters were set as follows: mass range for full-MS scan was m/z 200–2000 at a resolution of 120,000 using FTMS analyzer. The top seventh most abundant parent ions for each MS scan were selected for MS/MS spectra. The normalized collision energy for each MS/MS (CID) event using ITMS analyzer was set to 35%.

### Peptide mapping analysis

The raw data was processed and used for peptide mapping analysis using Biopharma Finder software version 3.0 (Thermo Scientific, Waltham, MA, USA). Protein sequences were added from amino acid 1–170 with a 6xHis tag, according to Q15116 (PDCD1_HUMAN) from the UniProt database. The parameters for variable modifications in peptide analysis were set as follows: the maximum number of modifications was set to 1, glycosylation was set to "Human", and side chain modifications included "Carbamidomethylation, Deamidation (N), Oxidation (MW)". The parameters for component detection in peptide mapping analysis were set as follows: the MS noise level was set to 4000, the S/N threshold was set to 32, and other parameters were set to their default values.

## Supplementary Information

Supplementary Figures.

## Data Availability

The datasets generated during and/or analyzed during the current study are available from the corresponding author on reasonable request.
