# Supplementary material for: Intact mass analysis reveals the novel O-linked glycosylation on the stalk region of PD-1 protein
Source: Sci Rep. 2023 Jun 14;13:9631. doi: 10.1038/s41598-023-36203-3 (PMC10267102; doi:10.1038/s41598-023-36203-3)
Supplement: Supplementary file 1 — Supplementary Figures. [file 41598_2023_36203_MOESM1_ESM.pdf]

SUPPLEMENTARY INFORMATION

Intact mass analysis reveals the novel O-linked glycosylation on the stalk region of PD-1 protein

Phanthakarn Tit-oon[1], Arisa Wonglangka[2], Klaichan Boonkanta[2], Mathuros Ruchirawat[1,3], Mayuree Fuangthong[1,3,4], Ram Sasisekharan[5,6], and Amnart Khongmanee[1,3,5,6*]

[1]Translational Research Unit, Chulabhorn Research Institute, Bangkok 10210, Thailand.

[2]Center for Biologics Research and Development, Chulabhorn Research Institute, Bangkok 10210, Thailand.

[3]Center of Excellence on Environmental Health and Toxicology (EHT), OPS, MHESI, Thailand.

[4]Program in Applied Biological Sciences, Chulabhorn Graduate Institute, Bangkok, 10210, Thailand.

[5]Koch Institute for Integrative Cancer Research, Massachusetts Institute of Technology, Cambridge, MA 02139, USA.

[6]Department of Biological Engineering, Massachusetts Institute of Technology, Cambridge, MA 02139, USA.

*To whom the correspondence should be addressed

Email: amnart@cri.or.th

SUPPLEMENTARY INFORMATION

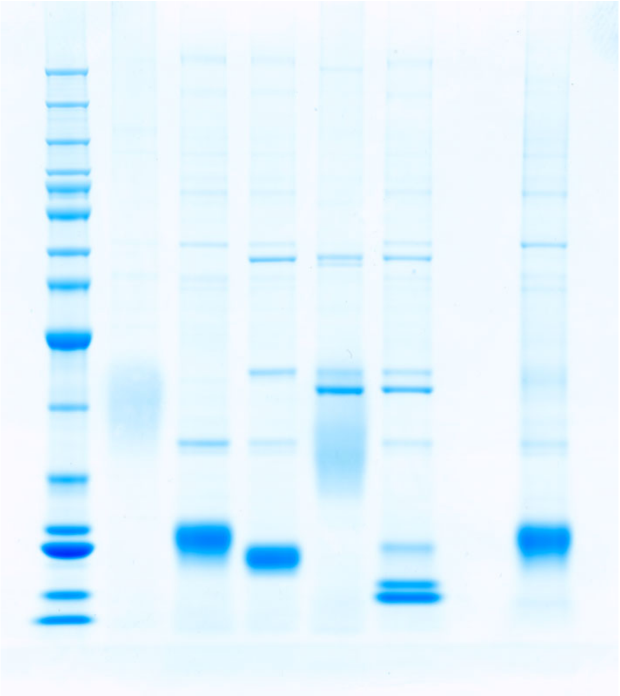

Supplementary Figure.S1 Uncropped SDS-PAGE of wild type PD-1 treated with various enzyme as shown in **Fig.1b**, a gel was scanned using EPSON Expression 11000XL scanner, SilverFast 8.0.1 r26 software.

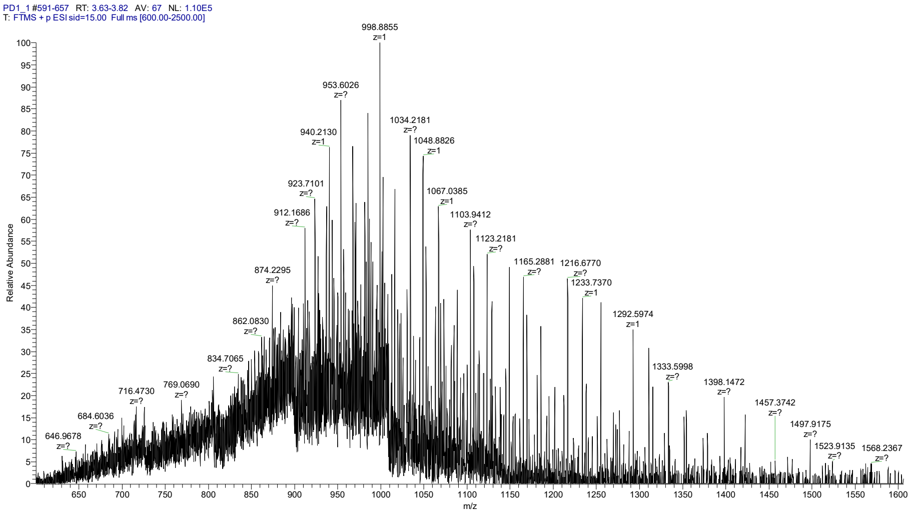

Supplementary Figure S2 Original image as shown in **Fig. 2a** that obtained from Qual Browser, Thermo Xcalibur 3.1.66.10 software. Mass spectrum average from the same range that used for protein deconvolution by Biopharma Finder software. Raw data from this spectrum were export without any modification and used for image reconstruction—for better clarity and flexibility—by GraphPad Prism software.

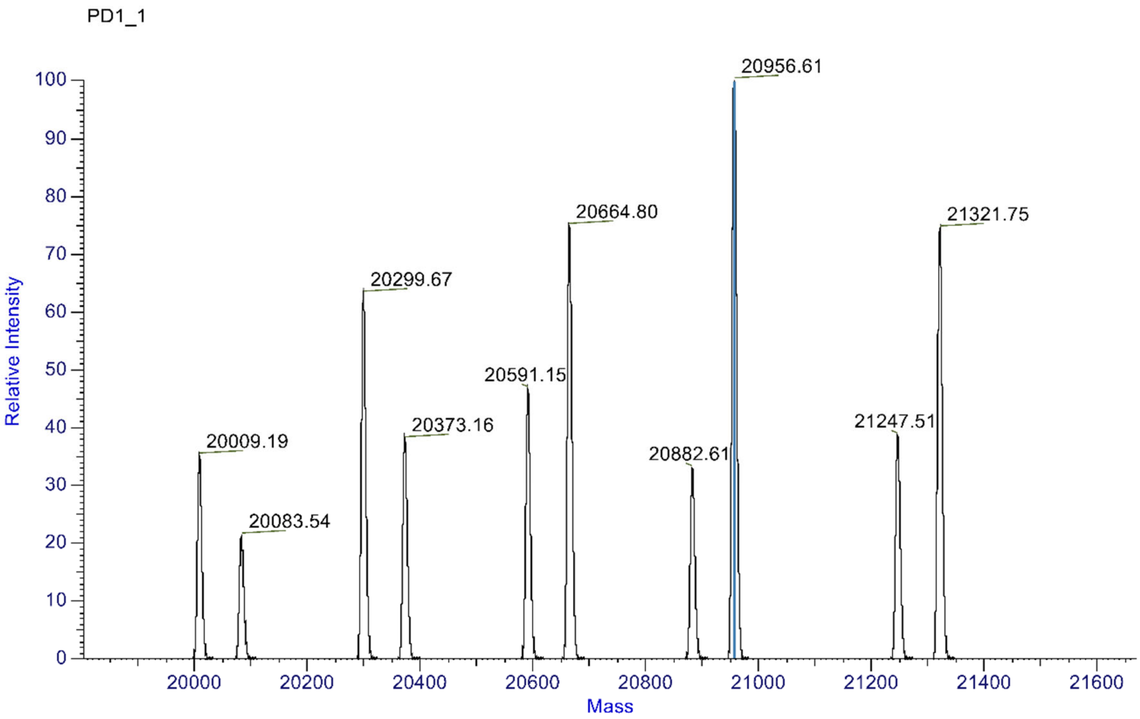

Supplementary Figure S3 Original image as shown in **Fig.2b** obtained from Biopharma Finder 3.0 software. Raw data from this deconvoluted mass spectrum were export without any modification and used for image reconstruction—for better clarity and flexibility—by GraphPad Prism software.

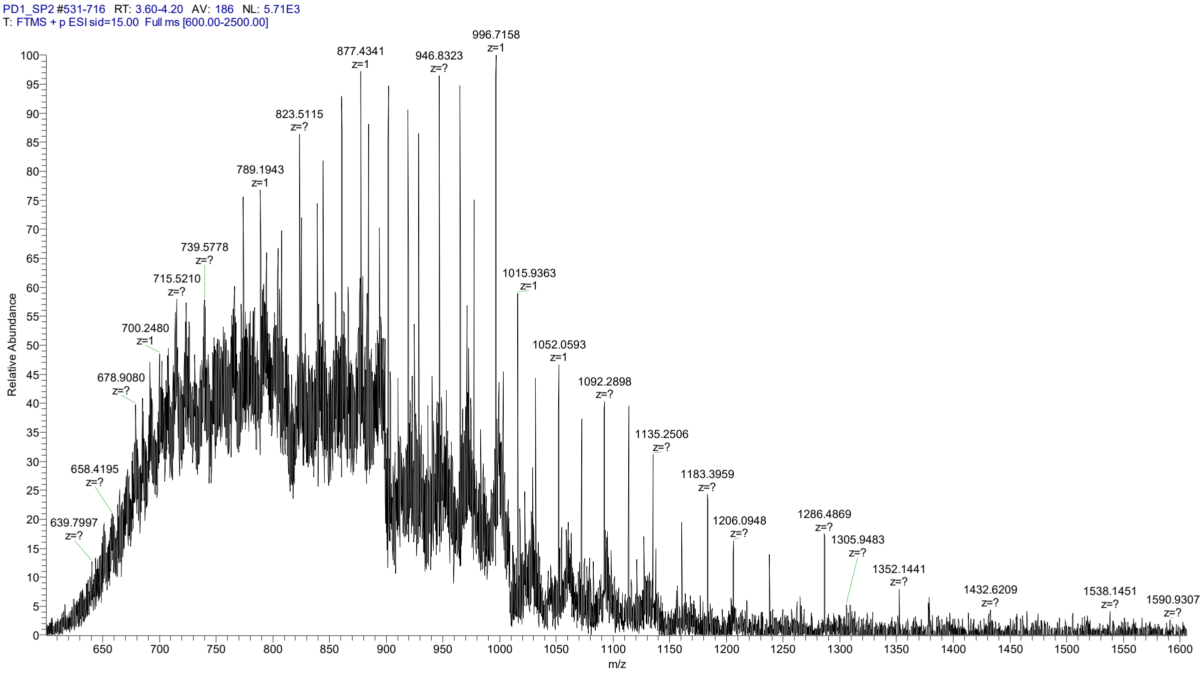

Supplementary Figure S4 Original image as shown in **Fig. 3a** that obtained from Qual Browser, Thermo Xcalibur 3.1.66.10 software. Mass spectrum average from the same range that used for protein deconvolution by Biopharma Finder software. Raw data from this spectrum were export without any modification and used for image reconstruction—for better clarity and flexibility—by GraphPad Prism software.

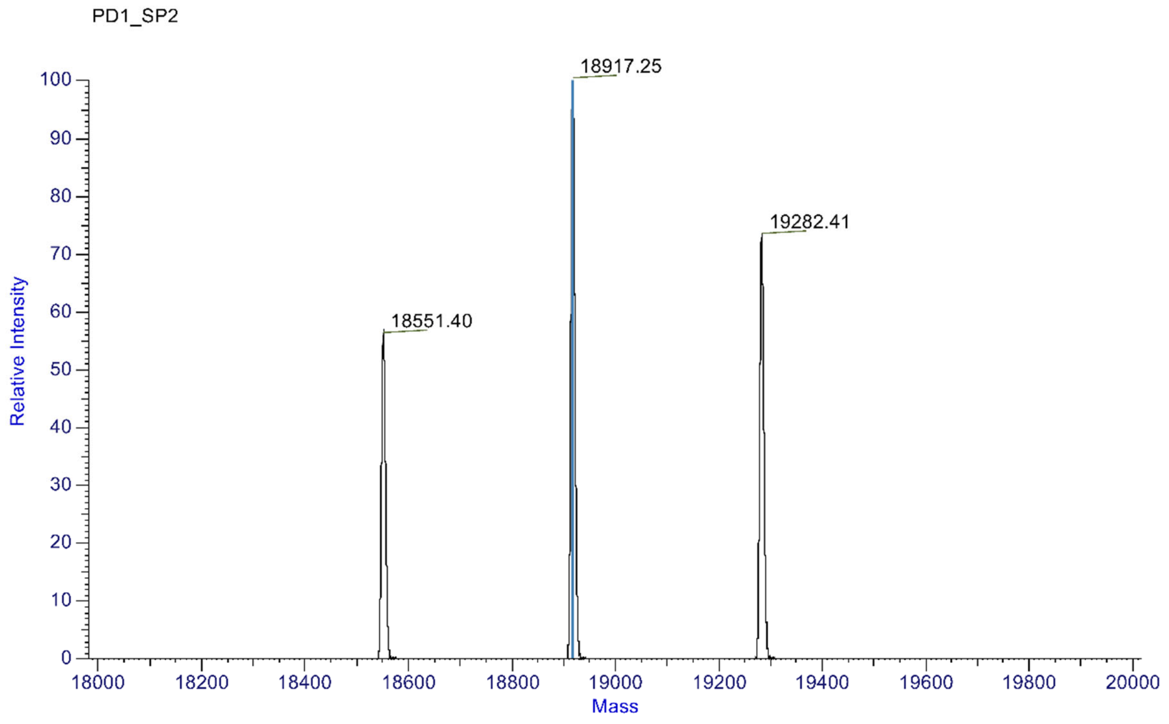

Supplementary Figure S5 Original image as shown in **Fig. 3b** obtained from Biopharma Finder 3.0 software. Raw data from this deconvoluted mass spectrum were export without any modification and used for image reconstruction—for better clarity and flexibility—by GraphPad Prism software.

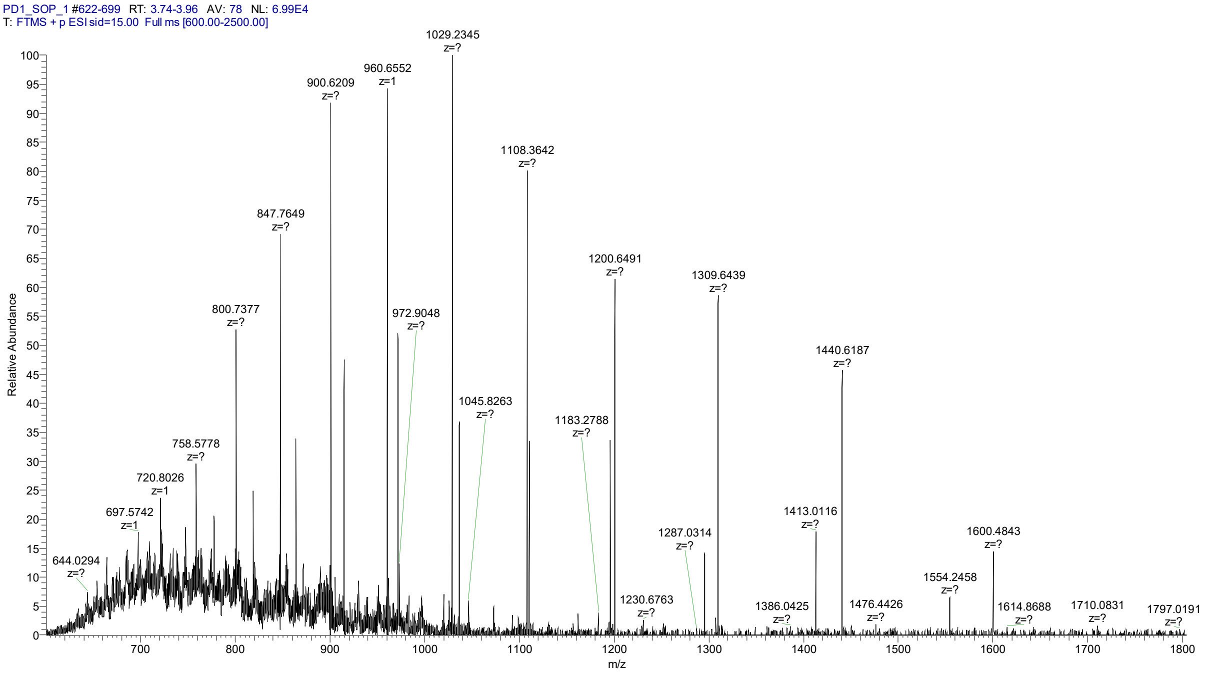

Supplementary Figure S6 Original image as shown in **Fig. 4a** that obtained from Qual Browser, Thermo Xcalibur 3.1.66.10 software. Mass spectrum average from the same range that used for protein deconvolution by Biopharma Finder software. Raw data from this spectrum were export without any modification and used for image reconstruction—for better clarity and flexibility—by GraphPad Prism software.

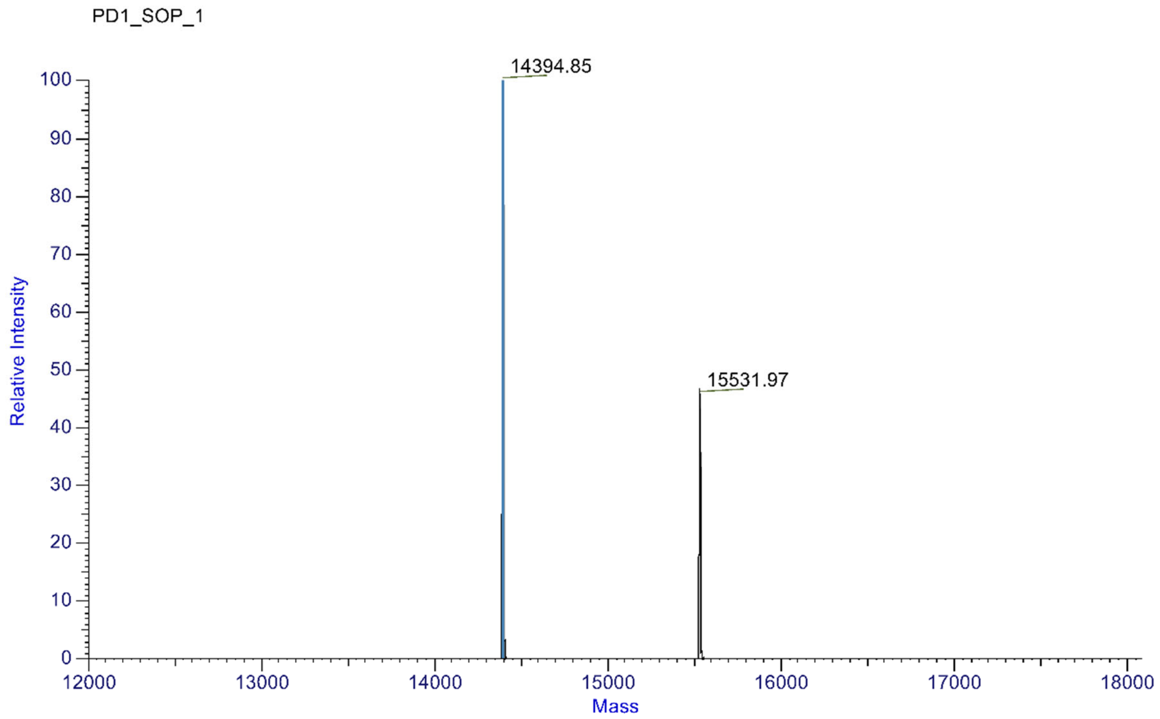

Supplementary Figure S7 Original image as shown in **Fig. 4b** obtained from Biopharma Finder 3.0 software. Raw data from this deconvoluted mass spectrum were export without any modification and used for image reconstruction—for better clarity and flexibility—by GraphPad Prism software.

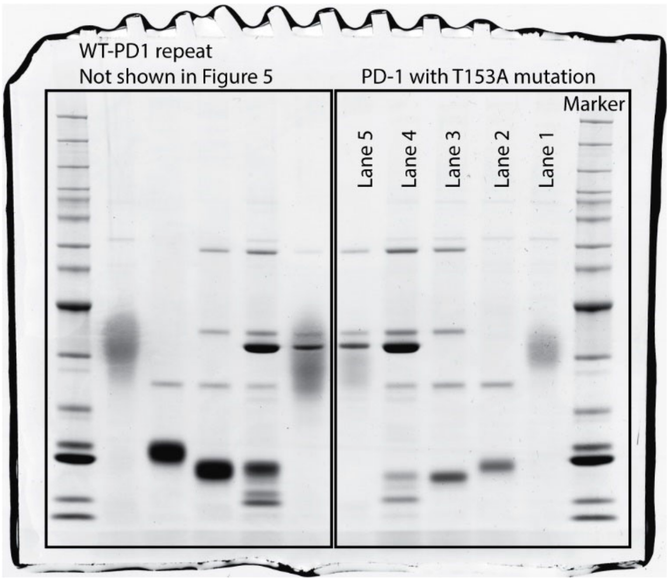

Supplementary Figure S8 Uncropped SDS-PAGE of PD-1 with T153A mutation treated with various enzyme as shown in **Fig.5a**, a gel was scanned using EPSON Expression 11000XL scanner, SilverFast 8.0.1 r26 software.

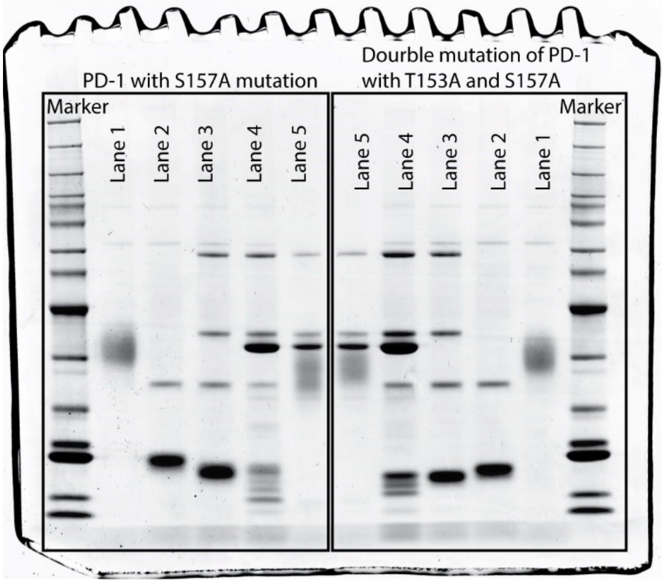

Supplementary Figure S9 Uncropped SDS-PAGE of PD-1 with T153A mutation treated with various enzyme as shown in **Fig.5b and 5c**, a gel was scanned using EPSON Expression 11000XL scanner, SilverFast 8.0.1 r26 software.

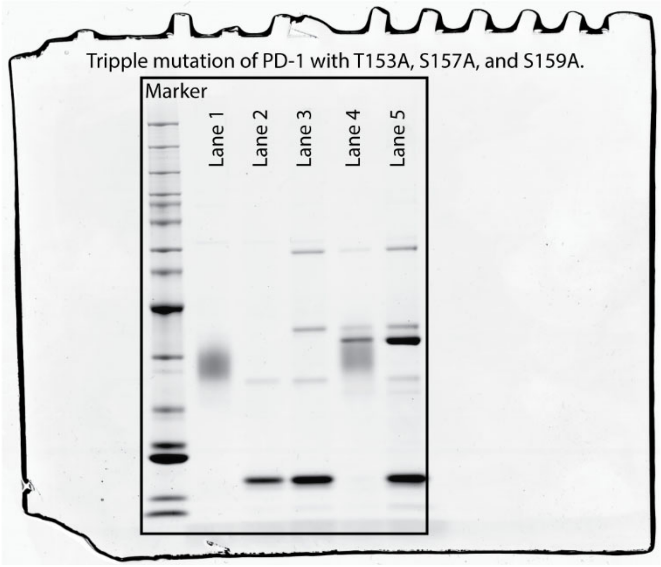

Supplementary Figure S10 Uncropped SDS-PAGE of PD-1 with T153A mutation treated with various enzyme as shown in **Fig.5d**, a gel was scanned using EPSON Expression 11000XL scanner, SilverFast 8.0.1 r26 software.

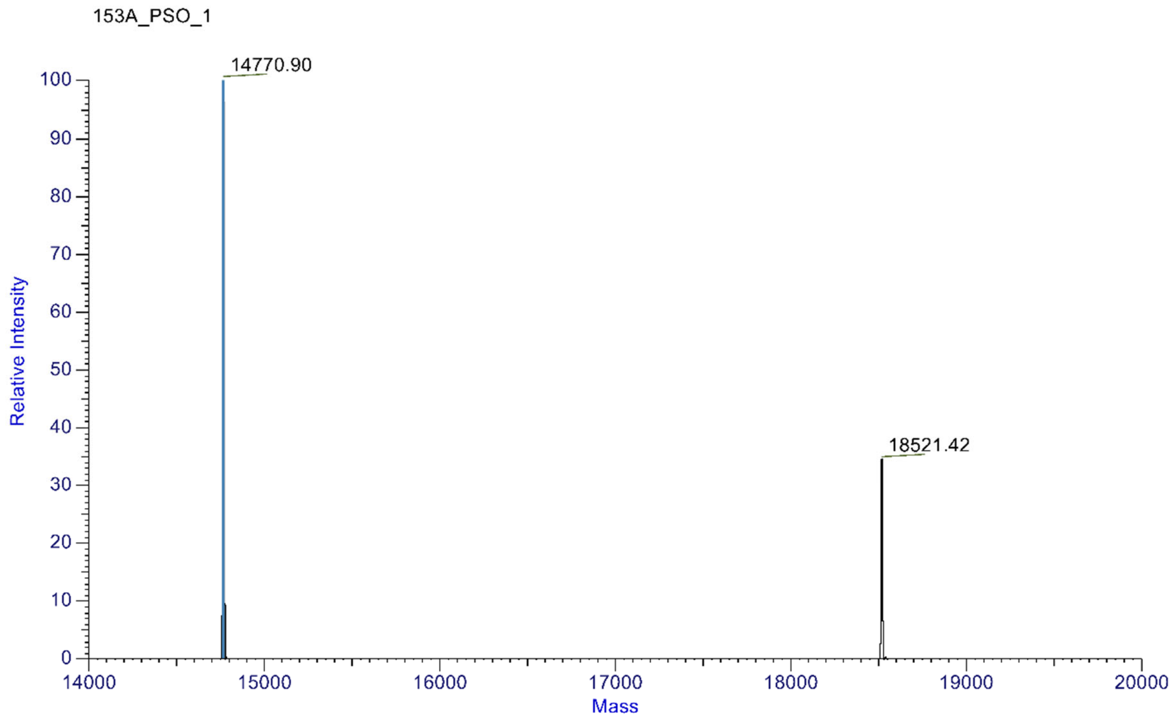

Supplementary Figure S11 Original image as shown in **Fig.6a** obtained from Biopharma Finder 3.0 software. Raw data from this deconvoluted mass spectrum were export without any modification and used for image reconstruction—for better clarity and flexibility—by GraphPad Prism software.

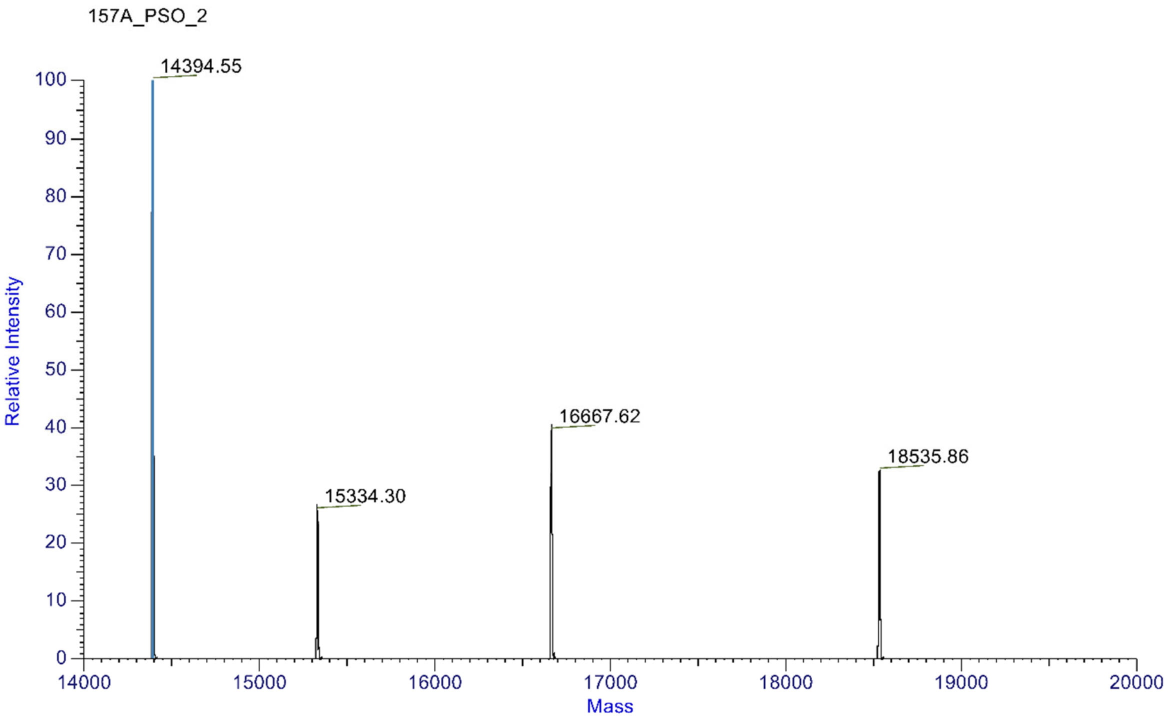

Supplementary Figure S12 Original image as shown in **Fig.6b** obtained from Biopharma Finder 3.0 software. Raw data from this deconvoluted mass spectrum were export without any modification and used for image reconstruction—for better clarity and flexibility—by GraphPad Prism software.

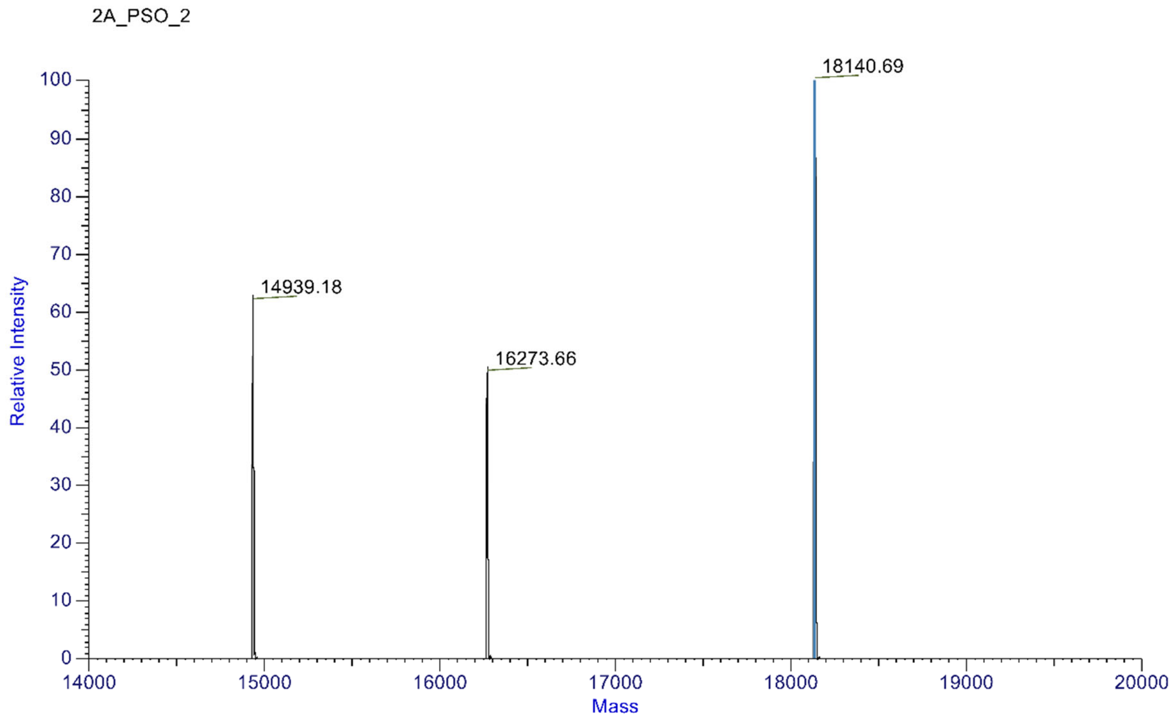

Supplementary Figure S13 Original image as shown in **Fig**.**6c** obtained from Biopharma Finder 3.0 software. Raw data from this deconvoluted mass spectrum were export without any modification and used for image reconstruction—for better clarity and flexibility—by GraphPad Prism software.

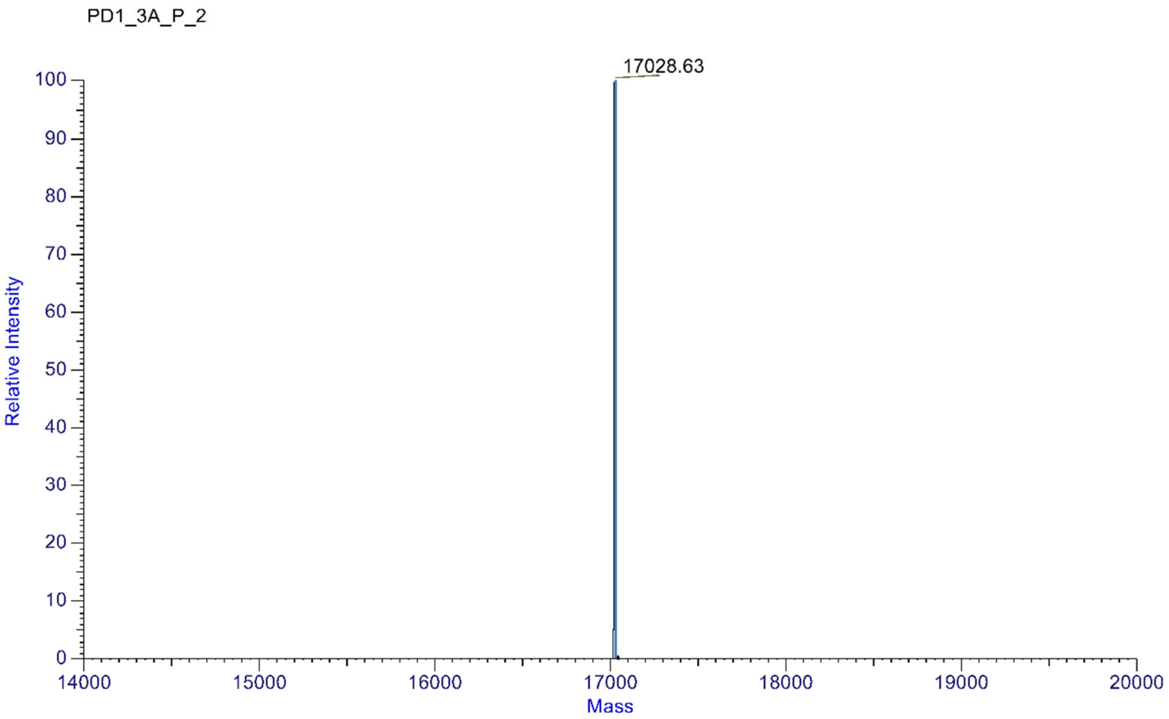

Supplementary Figure S14 Original image as shown in **Fig.6d** obtained from Biopharma Finder 3.0 software. Raw data from this deconvoluted mass spectrum were export without any modification and used for image reconstruction—for better clarity and flexibility—by GraphPad Prism software.

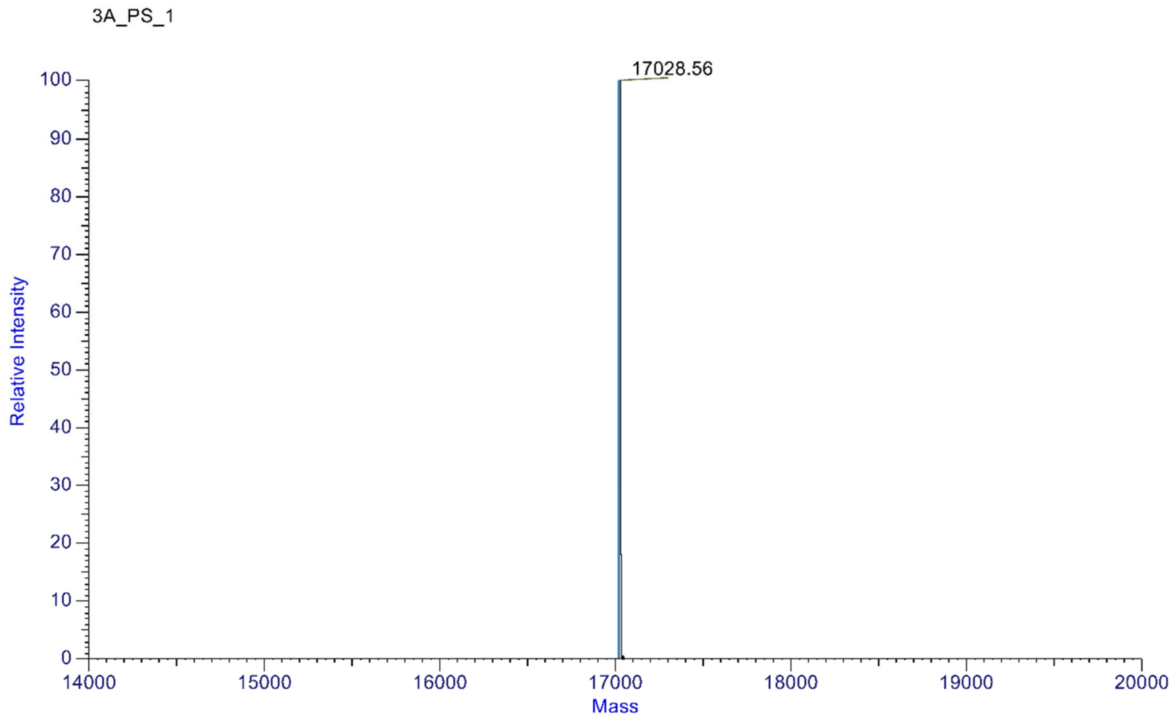

Supplementary Figure S15 Original image as shown in **Fig**.**6e** obtained from Biopharma Finder 3.0 software. Raw data from this deconvoluted mass spectrum were export without any modification and used for image reconstruction—for better clarity and flexibility—by GraphPad Prism software.

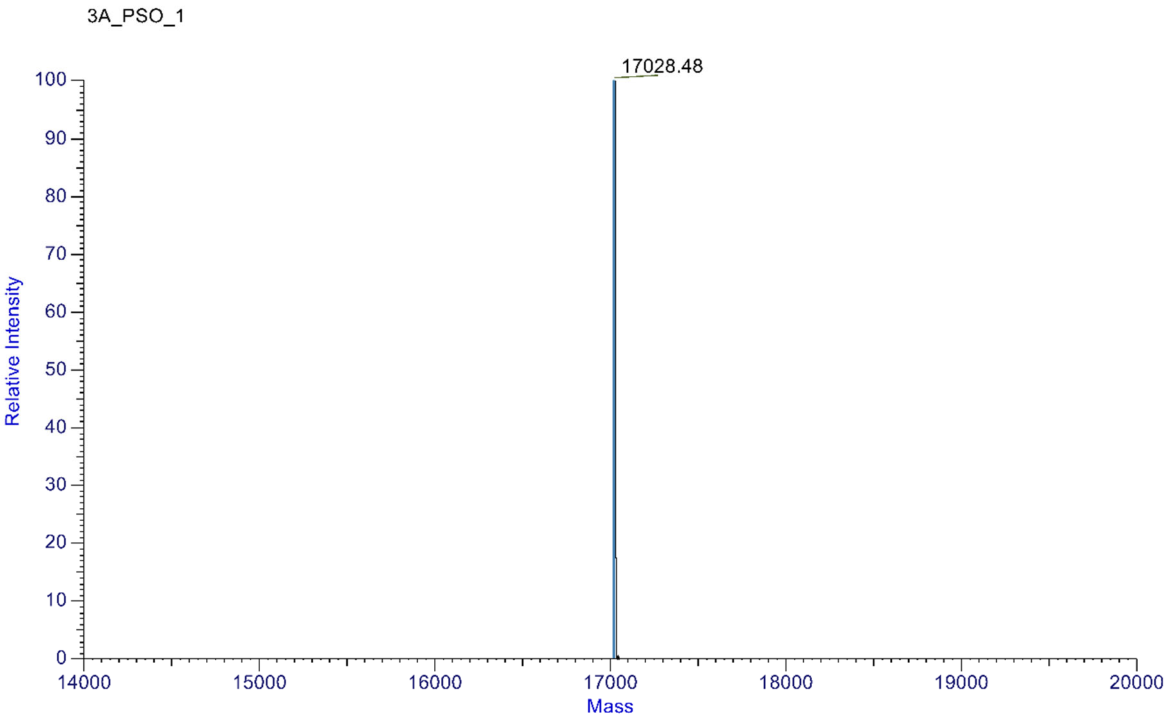

Supplementary Figure. **S16** Original image as shown in **Fig**. **6f** obtained from Biopharma Finder 3.0 software. Raw data from this deconvoluted mass spectrum were export without any modification and used for image reconstruction—for better clarity and flexibility—by GraphPad Prism software.

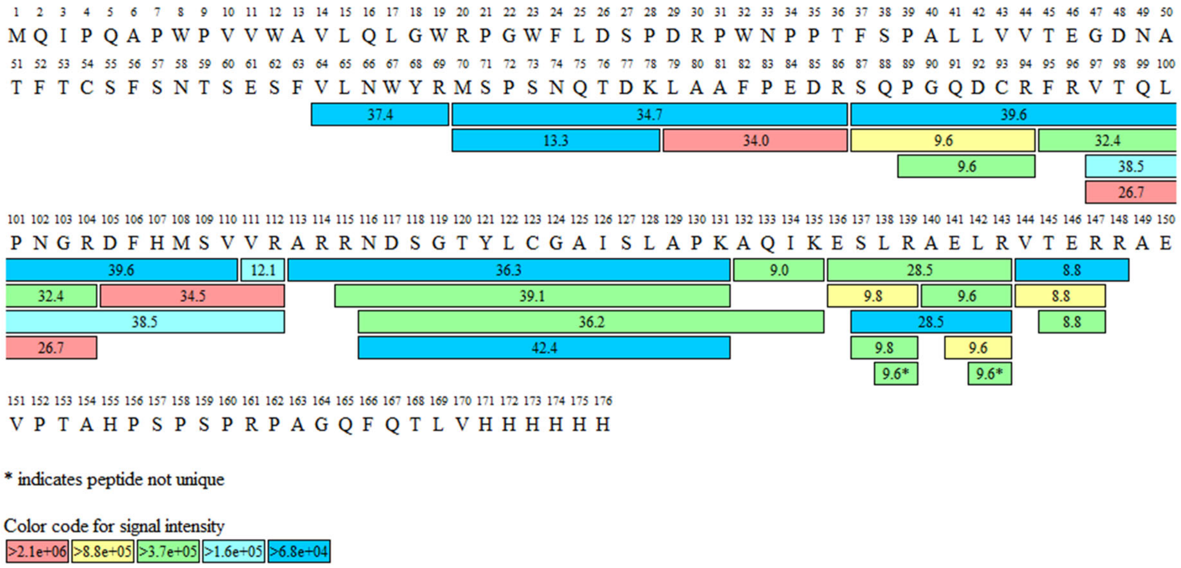

Supplementary Figure.S17 Sequence coverage map of MS/MS spectrum acquired from in-gel tryptic digestion of band a from Figure 1b, which corresponds to the untreated wild-type PD-1 protein. The coverage map was obtained using Biopharma Finder 3.0 software. It should be noted that no peptides related to OpeRATOR digestion were identified.

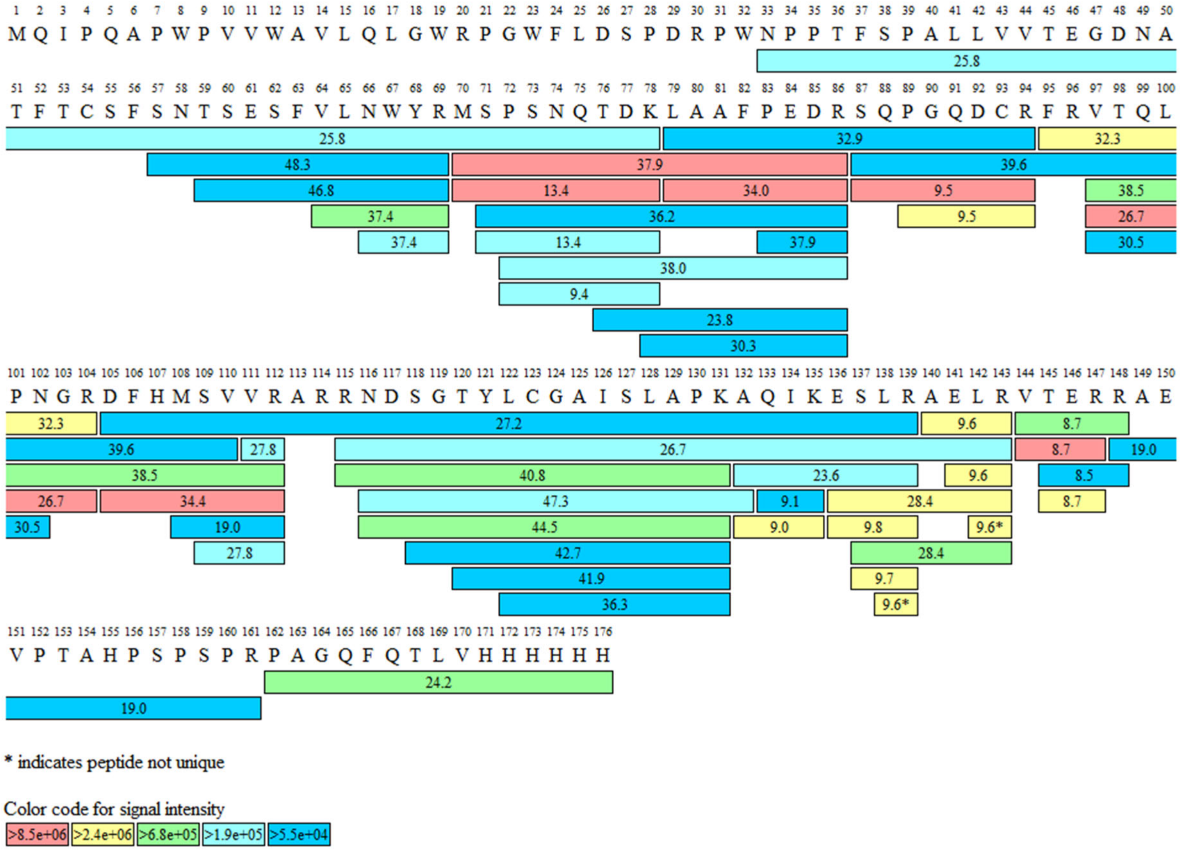

Supplementary Figure.S18 Sequence coverage map of MS/MS spectrum acquired from in-gel tryptic digestion of band b from Figure 1b, which corresponds to the PNGaseF treated wild-type PD-1 protein. The coverage map was obtained using Biopharma Finder 3.0 software. It should be noted that no peptides related to OpeRATOR digestion were identified.

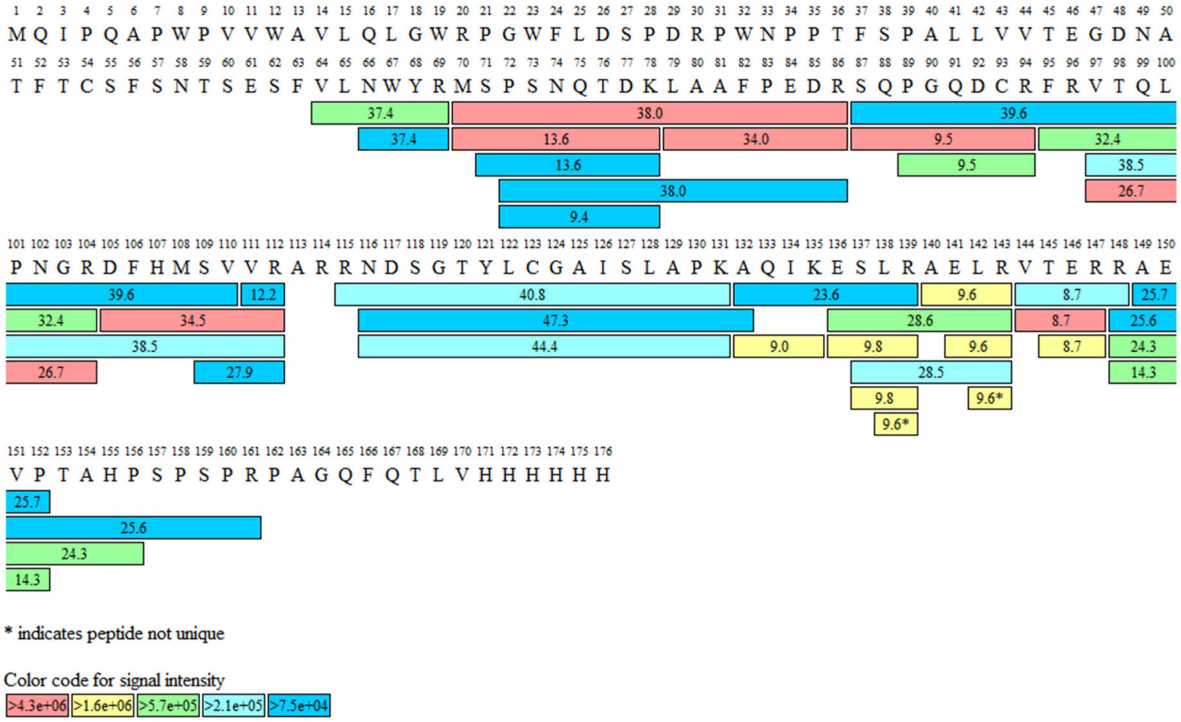

Supplementary Figure.S19 Sequence coverage map of MS/MS spectrum acquired from in-gel tryptic digestion of band d from Figure 1b, which corresponds to the OpeRATOR treated wild-type PD-1 protein (upper band). The coverage map was obtained using Biopharma Finder 3.0 software. It should be noted that peptides related to OpeRATOR digestion— with high abundance of RAEVPTHAP—were identified in this samples.

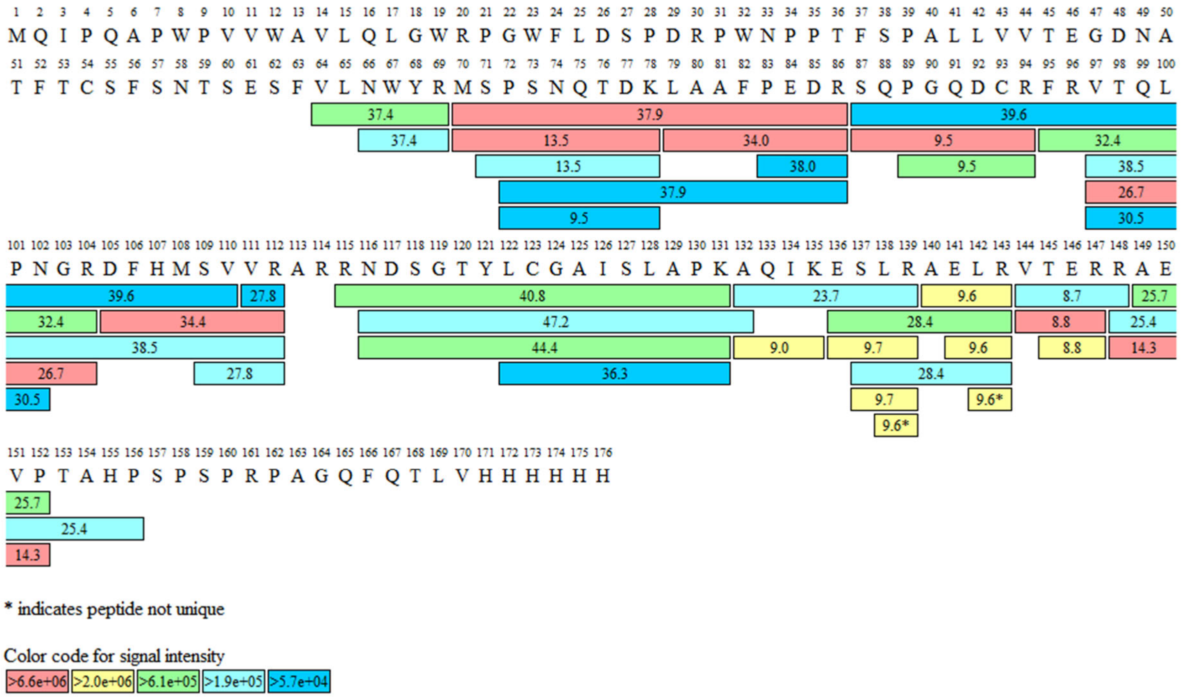

Supplementary Figure.S20 Sequence coverage map of MS/MS spectrum acquired from in-gel tryptic digestion of band e from Figure 1b, which corresponds to the OpeRATOR treated wild-type PD-1 protein (lower band). The coverage map was obtained using Biopharma Finder 3.0 software. It should be noted that peptides related to OpeRATOR digestion— with high abundance of RAEVP—were identified in this samples.

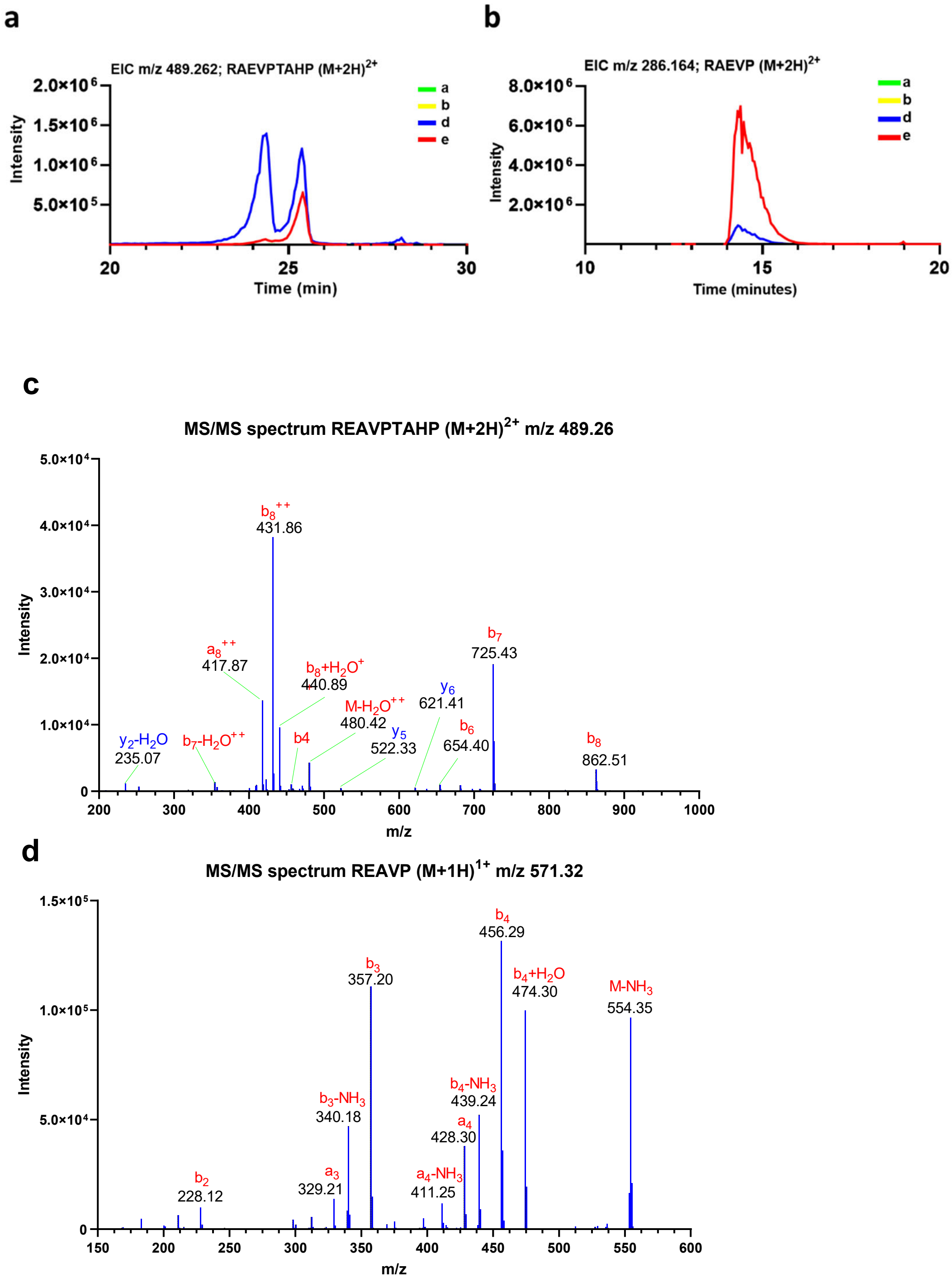

Supplementary Figure. S21 Extracted ion chromatogram and sequence confirmation of OpeRATOR- digested peptides. A) The EIC of m/ z 489. 262, which corresponds to RAEVPTAHP, was highly detected in the sample from band d, confirming that this fragment is a result of OpeRATOR digestion at S157. B) The EIC of m/z 286.164, which corresponds to RAEVP, was highly detected in the sample from band e, confirming that this fragment is a result of OpeRATOR digestion at T153. C) The b-ion and y-ion annotated MS/MS spectrum

corresponding to the parent ion with m/z 489.26 confirms the amino acid sequence of RAEVPTAHP. D) The b-ion and y-ion annotated MS/MS spectrum corresponding to the parent ion with m/z 571.32 confirms the amino acid sequence of RAEVP (note that MS/MS spectrum of m/z 286.16, corresponding to $(M+2H)^{2+}$, is also possible for confirming the sequence, but the spectrum of singlet charge parent ion quality is much better and was chosen to display).
